# Integrating Mendelian randomization and multi-omics analysis unravels gut microbiota-driven metabolic mechanisms in sepsis and identifies diagnostic biomarkers through experimental validation

**DOI:** 10.1063/5.0296018

**Published:** 2026-01-06

**Authors:** Guangyao Wang, Yuanyuan Liu, Jinjing Tan, Liqun Li, Jing Yan, Jing Liang, Xiaohua Hong, Sheng Xie

**Affiliations:** 1 Department of Respiratory and Critical Care Medicine, The First Affiliated Hospital of Guangxi University of Chinese Medicine, Nanning 530023, Guangxi, China; 2 Department of Center of Preventive Disease Treatment, The First Affiliated Hospital of Guangxi University of Chinese Medicine, Nanning 530023, Guangxi, China; 3 Department of Administration, The First Affiliated Hospital of Guangxi University of Chinese Medicine, Nanning 530023, Guangxi, China; 4 Department of Gastroenterology, The First Affiliated Hospital of Guangxi University of Chinese Medicine, Nanning 530023, Guangxi, China; 5 Department of Rehabilitation Medicine, The First Affiliated Hospital of Guangxi University of Chinese Medicine, Nanning 530023, China; 6 Guangxi Key Laboratory of Bioactive Molecules Research and Evaluation and College of Pharmacy, Guangxi Medical University, Nanning 530021, China; 7 Key Laboratory of Longevity and Aging-related Diseases of Chinese Ministry of Education and Center for Translational Medicine, Guangxi Medical University, Nanning 530021, China

## Abstract

Sepsis, a life-threatening systemic inflammatory syndrome, remains a leading cause of global mortality due to its complex pathophysiology and the lack of specific diagnostic biomarkers. Recent evidence highlights intricate interactions between the gut microbiota, metabolites, and host inflammatory responses; however, the causal relationships and underlying mechanisms remain poorly understood. We integrated Mendelian randomization (MR) with multi-omics approaches (including transcriptomics, untargeted metabolomics, and single-cell transcriptomics) to elucidate the causal relationships and underlying mechanisms between gut microbiota and their associated metabolites in the inflammatory response of sepsis. Building on this analysis, we employed machine learning algorithms to identify sepsis-specific diagnostic biomarkers derived from *Prevotella 9*, fatty acids, and PANoptosis-related genes. These key diagnostic genes were experimentally validated using a pulmonary sepsis organoid model. Seven gut microbiota taxa were identified as causally associated with sepsis, with *Prevotella 9* demonstrating significant protective effects (odds ratio = 0.89, *P* = 0.01). The protective role of *Prevotella 9* appears to be mediated through its regulation of fatty acid metabolism. Machine learning algorithms pinpointed three key diagnostic genes for sepsis: *ABCC1*, *CYP1B1*, and *PPARG*. Validation in an independent cohort (area under the receiver operating characteristic curve = 0.93) and the lung-derived organoid model confirmed their relevance. Functional analyses revealed that these genes are involved in immunometabolic pathways, including neutrophil regulation, oxidative stress, and macrophage polarization, and are predominantly expressed in monocytes. This study integrates MR and multi-omics analyses to reveal that *Prevotella 9* may regulate sepsis through lipid metabolism. Additionally, three key genes (*ABCC1*, *CYP1B1*, and *PPARG*) were identified based on *Prevotella 9*, fatty acids, and PANoptosis, contributing to sepsis progression via the regulation of neutrophils, oxidative stress, and macrophage polarization. Monocytes may serve as potential cellular targets for sepsis.

## I. INTRODUCTION

Sepsis is a systemic inflammatory response syndrome triggered by pathogenic infections, such as viruses and bacteria. It results in the dysfunction of multiple organ systems, often culminating in life-threatening outcomes.^1^ Despite significant advancements in treatment strategies, the mortality rate of sepsis remains alarmingly high.^2,3^ As one of the leading causes of death among critically ill patients worldwide, this high mortality is primarily attributed to the complex pathophysiological mechanisms of sepsis and the absence of specific diagnostic biomarkers. Consequently, there is an urgent need to deepen our understanding of sepsis pathophysiology and to identify novel diagnostic and therapeutic approaches.

Recent studies have highlighted the intricate interactions between gut microbiota, metabolites, and host inflammatory responses.^4,5^ However, traditional observational studies face substantial limitations in elucidating the causal relationships and underlying mechanisms among these factors. While genome-wide association studies (GWAS) have enabled the identification of genetic risk factors, integrating multi-omics data to decipher the regulatory axis of gut microbiota–metabolites–sepsis remains a formidable challenge. Mendelian randomization (MR) analysis leverages genetic variations as instrumental variables (IVs) to infer causality between exposures and outcomes. Since genetic variants are randomly allocated at conception and fixed throughout life, they are generally unaffected by environmental confounders or reverse causation. This “natural randomization” mimics randomized controlled trials, allowing causal inference from observational data. MR requires three key assumptions: genetic variants must be associated with the exposure (relevance), independent of confounders (independence), and affect the outcome only through the exposure (exclusion restriction).^6^ Since alleles are randomly allocated at conception, MR effectively minimizes confounding factors,^7^ making it a robust and reliable method for establishing causality. Previous MR studies have established causal links between gut microbiota and sepsis risk. Shang *et al.*^8^ and Zhang *et al.*^9^ reported that Lentisphaerae showed protective associations with sepsis [odds ratio (OR) = 0.89 and 0.93, respectively], while Yang *et al.*^10^ identified Bacteroidales as a risk factor (OR = 1.49). However, these studies had three key limitations: (I) they focused solely on establishing causal relationships without exploring underlying mechanisms; (II) they did not investigate metabolic mediators; and (III) they lacked experimental validation. The molecular mechanisms underlying the interactions between gut microbiota, their metabolites, and the inflammatory response in sepsis remain poorly understood and require further exploration.

This study aims to systematically investigate the causal relationship between gut microbiota and sepsis using MR analysis while elucidating the metabolic pathways that mediate this relationship. By integrating multi-omics datasets, we seek to comprehensively uncover the mechanisms through which gut microbiota and their metabolites influence the inflammatory response in sepsis. A machine learning-based approach will be employed to identify sepsis-specific diagnostic biomarkers from the multi-omics data. Furthermore, this study introduces a novel lung-derived sepsis organoid model to validate the identified diagnostic genes. This organoid model effectively mimics the *in vivo* organ microenvironment, providing an ideal platform for biomarker validation.^11^ In previous work by our research group, we demonstrated that in a sepsis rat model induced by lipopolysaccharide combined with *Staphylococcus aureus* and *Escherichia coli*, key genes associated with PANoptosis were significantly dysregulated in lung tissues. PANoptosis, a unique form of cell death that integrates pyroptosis, apoptosis, and necroptosis, has been shown to play a critical role in inflammation regulation and disease progression.^12^ This study explores the mechanisms underlying the interactions between gut microbiota, their associated metabolites, and the inflammatory response in sepsis, with a specific focus on PANoptosis. By unraveling these mechanisms, we aim to lay a foundation for personalized therapeutic strategies targeting sepsis.

## II. RESULTS

### A. MR analysis of gut microbiota genera and sepsis

**FIG. 1.**
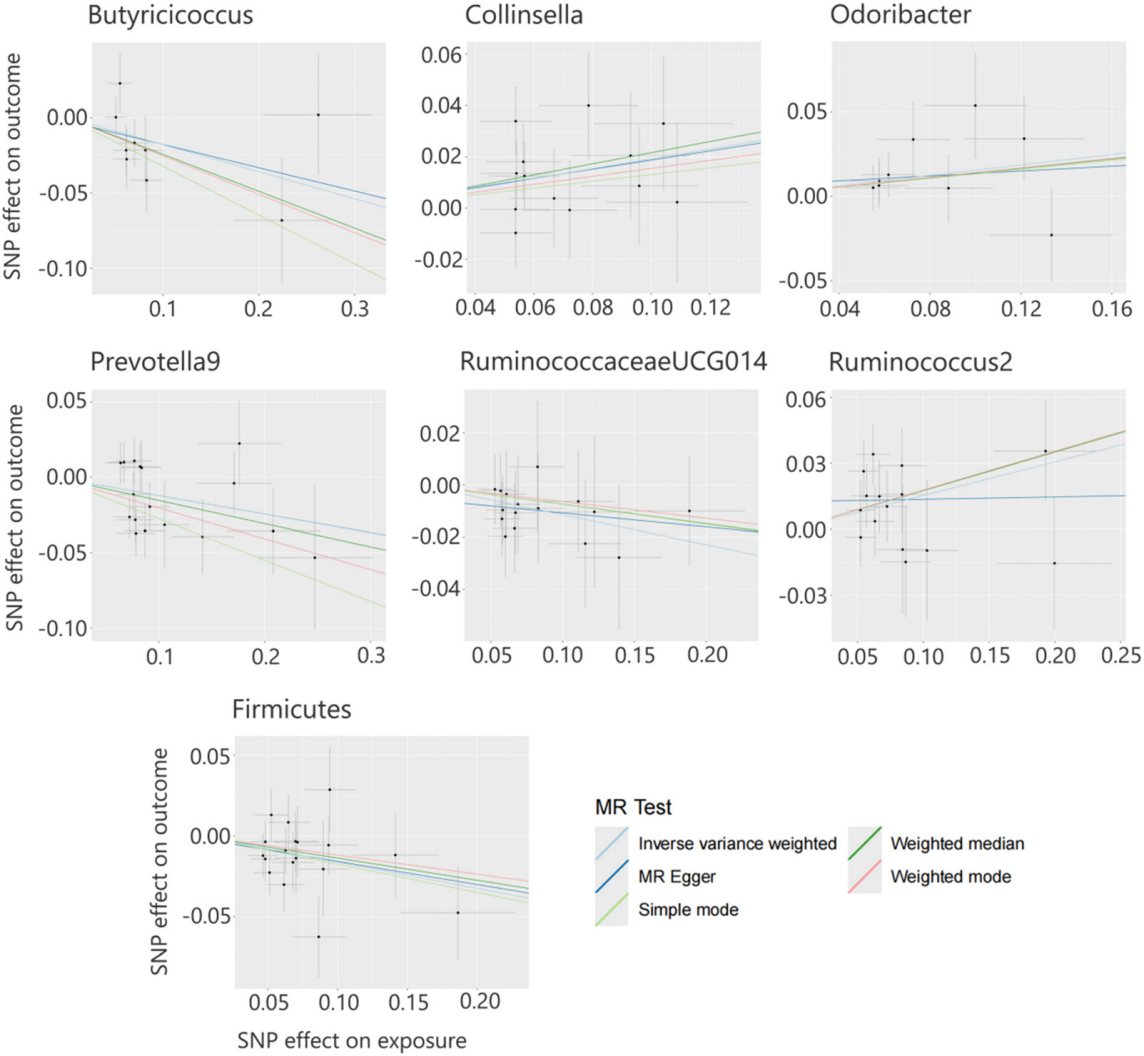
Scatter plot showing the causal relationship between seven types of gut microbiota and sepsis.

**FIG. 2.**
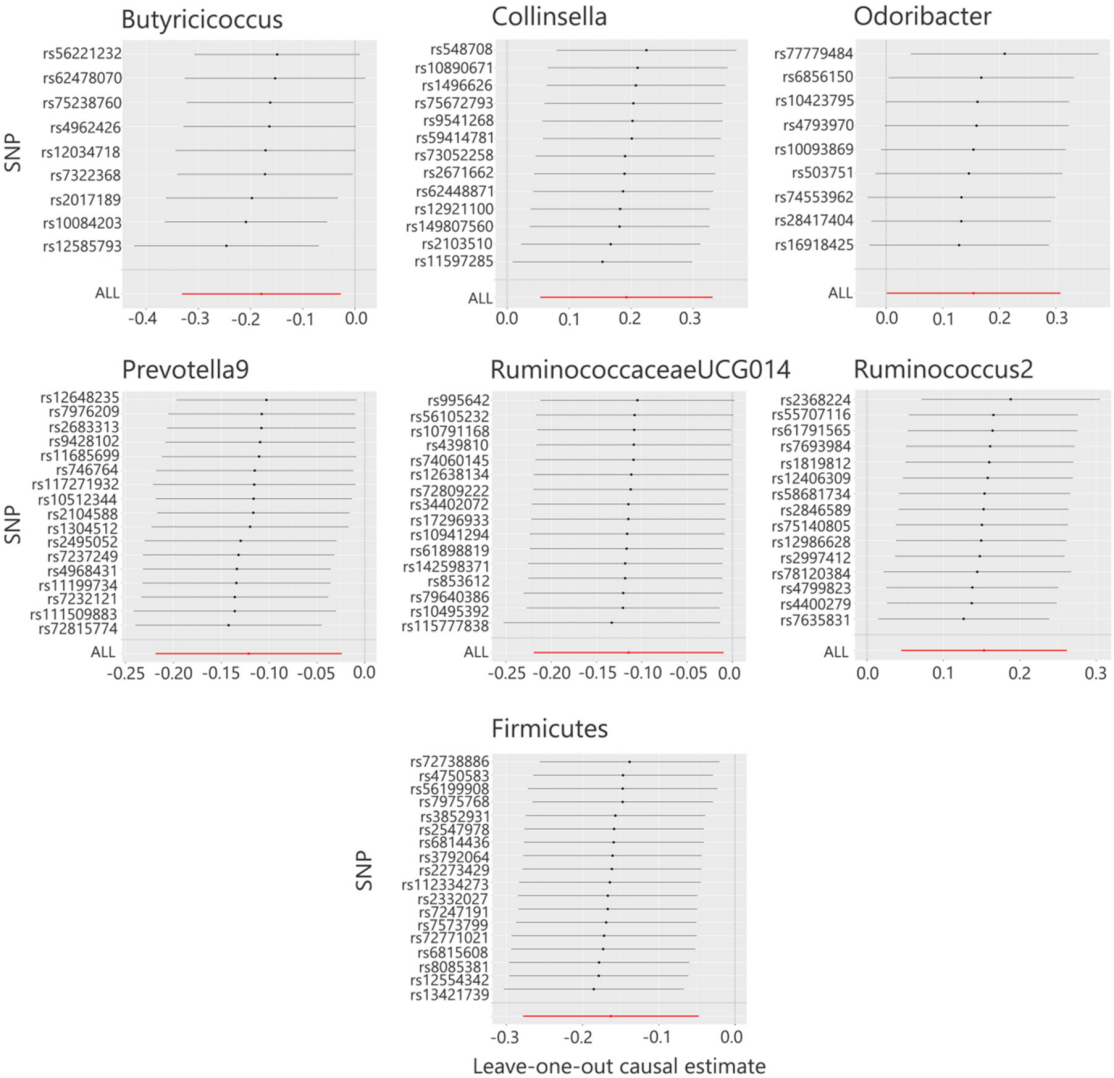
MR leave-one-out sensitivity analyses of the causal relationship between seven types of gut microbiota and sepsis.

Based on the selection criteria for instrumental variables (IVs), 1232 single-nucleotide polymorphisms (SNPs) were identified as IVs for 119 gut microbiota genera. The MR analysis (Table I, Figs. 1 and 2) identified seven genera (*Butyricicoccus*, *Collinsella*, *Odoribacter*, *Prevotella 9*, *Ruminococcaceae UCG014*, *Ruminococcus 2*, and *Firmicutes*) that were significantly associated with sepsis. Using the inverse variance weighted (IVW) method, recognized for its robustness in causal inference, *Butyricicoccus* was found to have a protective effect against sepsis [OR = 0.84, 95% confidence interval (CI): 0.62–1.19, *P* = 0.02], with consistent findings from the weighted median (WM) method (OR = 0.78, 95% CI: 0.64–0.96, *P* = 0.02). Similarly, *Prevotella 9* (an unassigned clade within the *Prevotella* genus that represents a distinct taxonomic group pending formal species designation) exhibited a protective effect in the IVW analysis (OR = 0.89, 95% CI: 0.80–0.98, *P* = 0.01), corroborated by the WME and simple mode (SM) methods. The IVW analysis also demonstrated protective effects for *Ruminococcaceae UCG014* and *Firmicutes* (OR < 1, *P* < 0.05); however, these associations were not supported by the other four evaluation methods. Conversely, *Collinsella* (OR = 1.21, 95% CI: 1.05–1.39, *P* = 0.01) and *Ruminococcus 2* (OR = 1.17, 95% CI: 1.05–1.30, *P* = 0.01) were identified as risk factors for sepsis, with the WME method supporting both findings. Additionally, the IVW method suggested *Odoribacter* as a risk factor (OR = 1.17, 95% CI: 1.00–1.36, *P* = 0.01).

**TABLE I.** Seven types of gut microbiota that are significantly associated with sepsis. OR: odds ratio; CI: confidence interval.

Bacterial taxa (exposure)	MR method	No. of SNP	*F*-statistic	OR	95% CI	*P*-value	Heterogeneity
*Butyricicoccus* genus.id.2055	IVW	9	>10	0.84	0.62–1.19	0.02	0.43
	MR-Egger	9		0.86	0.62–1.19	0.39	
	Weighted median	9		0.78	0.64–0.96	0.02	
	Weighted mode	9		0.78	0.57–1.05	0.14	
	Simple mode	9		0.72	0.52–1.01	0.09	
*Collinsella* genus.id.815	IVW	13	>10	1.21	1.05–1.39	0.01	0.67
	MR-Egger	13		1.20	0.67–2.13	0.56	
	Weighted median	13		1.24	1.02–1.51	0.03	
	Weighted mode	13		1.17	0.85–1.60	0.35	
	Simple mode	13		1.14	0.84–1.54	0.41	
*Odoribacter* genus.id.952	IVW	9	>10	1.17	1.00–1.36	0.05	0.67
	MR-Egger	9		1.08	0.64–1.80	0.79	
	Weighted median	9		1.15	0.94–1.40	0.17	
	Weighted mode	9		1.15	0.85–1.55	0.39	
	Simple mode	9		1.14	0.83–1.56	0.43	
*Prevotella 9* genus.id.11183	IVW	17	>10	0.89	0.80–0.98	0.01	0.23
	MR-Egger	17		0.89	0.67–1.16	0.40	
	Weighted median	17		0.86	0.75–0.98	0.02	
	Weighted mode	17		0.82	0.59–0.98	0.08	
	Simple mode	17		0.76	0.66–1.01	0.05	
*RuminococcaceaeUCG014* genus.id.11371	IVW	16	>10	0.89	0.80–0.99	0.03	1.00
	MR-Egger	16		0.95	0.73–1.24	0.70	
	Weighted median	16		0.93	0.81–1.07	0.29	
	Weighted mode	16		0.94	0.77–1.14	0.53	
	Simple mode	16		0.93	0.74–1.16	0.52	
*Ruminococcus 2* genus.id.11374	IVW	15	>10	1.17	1.05–1.30	0.01	0.62
	MR-Egger	15		1.01	0.78–1.31	0.94	
	Weighted median	15		1.19	1.02–1.39	0.03	
	Weighted mode	15		1.19	0.95–1.51	0.16	
	Simple mode	15		1.19	0.92–1.56	0.21	
*Firmicutes* Phylum.id.1672	IVW	18	>10	0.85	0.76–0.95	0.01	0.66
	MR-Egger	18		0.87	0.63–1.19	0.39	
	Weighted median	18		0.87	0.74–1.02	0.09	
	Weighted mode	18		0.89	0.71–1.11	0.32	
	Simple mode	18		0.84	0.63–1.11	0.24	

All seven causal associations had *F*-statistics greater than 10, minimizing the risk of weak IV bias. Cochran's Q test results revealed no significant heterogeneity (*P* > 0.05), while scatter plots (Fig. 1) and leave-one-out plots (Fig. 2) indicated no significant outliers. Furthermore, MR-Egger regression intercept analysis found no evidence of horizontal pleiotropy (*P* > 0.05; supplementary material Table S1). These findings suggest that the MR analysis of gut microbiota genera and sepsis in this study is robust and reliable.

### B. MR analysis of *Prevotella 9* and metabolites

**FIG. 3.**
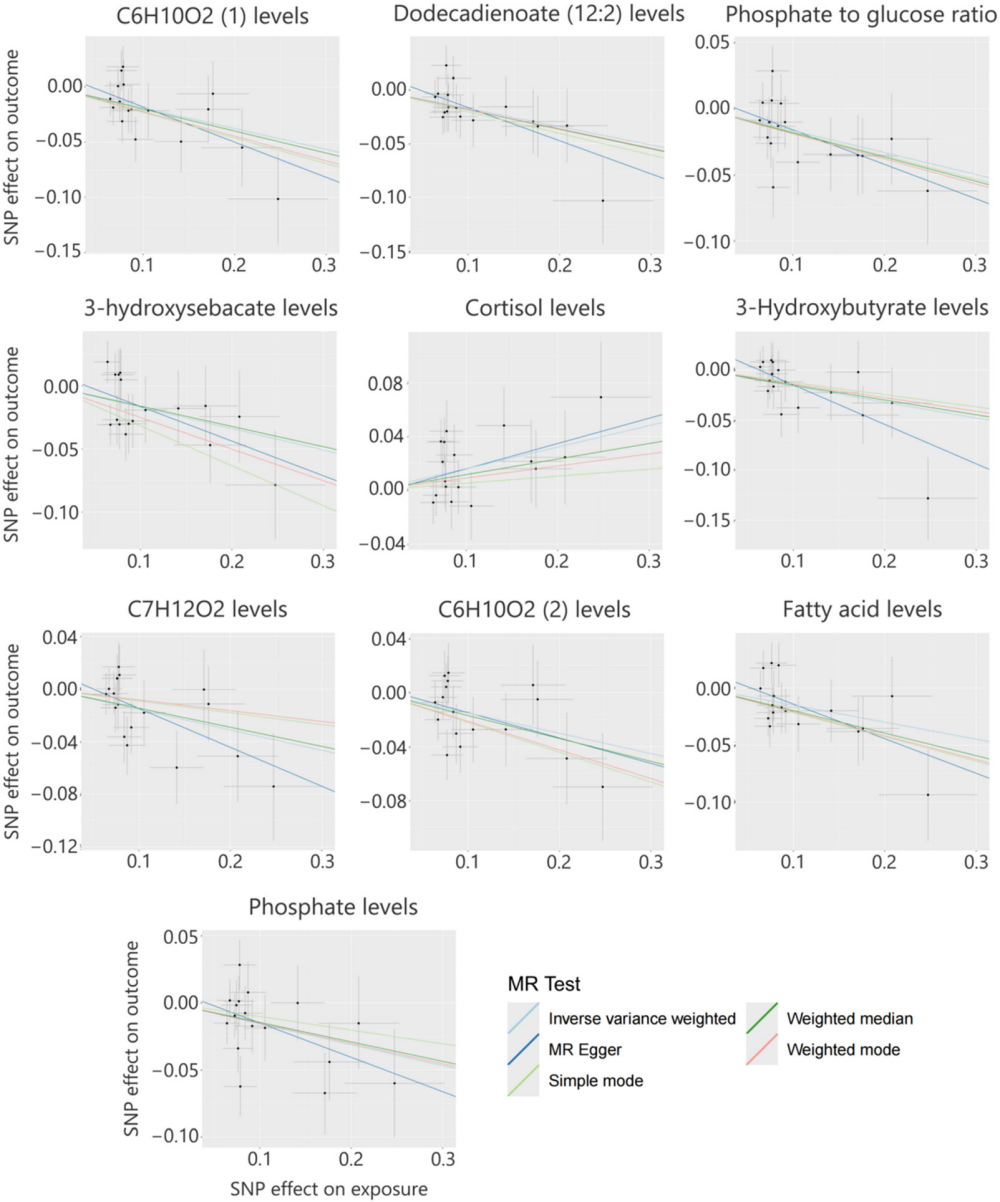
Scatter plot showing the causal relationship between the top 10 metabolites and *Prevotella 9.*

**FIG. 4.**
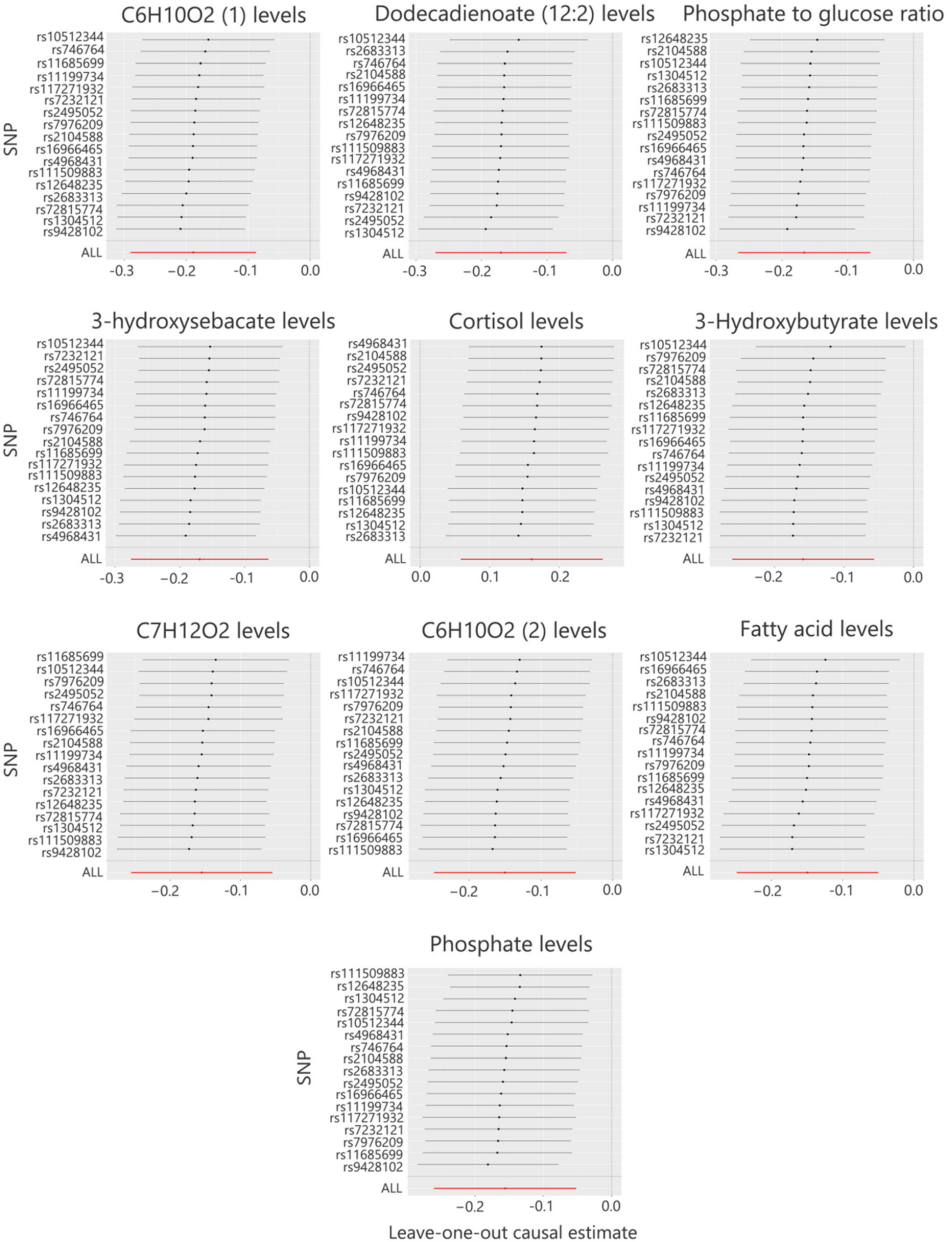
MR leave-one-out sensitivity analyses of the causal relationship between the top 10 metabolites and *Prevotella 9.*

The MR analysis of gut microbiota genera and sepsis revealed a significant causal association between *Prevotella 9* and sepsis across multiple evaluation methods. Previous studies^13^ have demonstrated that gut microbiota influence disease onset and progression through the modulation of metabolites. Building on these findings, this study employed MR analysis to identify metabolites causally associated with *Prevotella 9*. The MR analysis identified 122 metabolites, including 17 unidentified metabolites, that were causally associated with *Prevotella 9* (supplementary material Table S2). This study focuses on the top 10 known metabolites that exhibited significant causal associations with *Prevotella 9* (Table II). Using the IVW method, a positive association was identified between *Prevotella 9* and plasma cortisol levels (OR = 1.17, 95% CI: 1.06–1.30, *P* < 0.001). In contrast, nine other metabolites displayed negative associations with *Prevotella 9*, as summarized below: (1) Glutamine conjugate of C_6_H_10_O_2_: OR = 0.83, 95% CI: 0.75–0.92, *P* < 0.001 (confirmed by MR-Egger and weighted median methods). (2) Dodecadienoate (12:2): OR = 0.84, 95% CI: 0.76–0.93, *P* < 0.001 (confirmed by MR-Egger and weighted median methods). (3) Phosphate-to-glucose ratio: OR = 0.85, 95% CI: 0.77–0.94, *P* < 0.001 (supported by the weighted median method). (4) 3-Hydroxysebacate: OR = 0.84, 95% CI: 0.76–0.94, *P* < 0.001 (supported by weighted median and simple mode methods). (5) 3-Hydroxybutyrate: OR = 0.85, 95% CI: 0.77–0.94, *P* < 0.001 (confirmed by MR-Egger and weighted median methods). (6) Glutamine conjugate of C_7_H_12_O_2_: OR = 0.86, 95% CI: 0.77–0.95, *P* < 0.001 (confirmed by MR-Egger and weighted median methods). (7) Glutamine conjugate of C_6_H_10_O_2_ (2): OR = 0.86, 95% CI: 0.78–0.95, *P* < 0.001 (supported by the weighted median method). (8) Branched-chain, straight-chain, or cyclopropyl 12:1 fatty acid: OR = 0.86, 95% CI: 0.78–0.95, *P* < 0.001 (confirmed by MR-Egger and weighted median methods). (9) Phosphate: OR = 0.86, 95% CI: 0.77–0.95, *P* < 0.001 (supported by the weighted median method). The MR analysis showed no evidence of heterogeneity or horizontal pleiotropy, as indicated by Cochran's Q test, MR-Egger regression, and I^2^ (supplementary material Table S4). Scatter plots (Fig. 3) and leave-one-out plots (Fig. 4) indicated no significant outliers. These findings suggest that the results are robust and reliable.

**TABLE II.** The top 10 metabolites causally related to *Prevotella 9*. GCST90200176: glutamine conjugate of C_6_H_10_O_2_ (1) levels; GCST90200190: dodecadienoate (12:2) levels; GCST90200767: phosphate-to-glucose ratio; GCST90199704: 3-hydroxysebacate levels; GCST90200378: cortisol levels (plasma); GCST90200367: 3-hydroxybutyrate levels; GCST90200167: glutamine conjugate of C_7_H_12_O_2_ levels; GCST90200173: glutamine conjugate of C_6_H_10_O_2_ (2) levels; GCST90200249: branched-chain, straight-chain, or cyclopropyl 12:1 fatty acid levels; and GCST90200393: phosphate levels.

No.	Metabolites (exposure)	MR method	No. of SNP	*F*-statistic	OR	95% CI	*P*-value	Heterogeneity
1	GCST90200176	IVW	17	>10	0.83	0.75–0.92	0.00	0.59
		MR-Egger	17		0.73	0.56–0.95	0.03	
		Weighted median	17		0.82	0.71–0.95	0.01	
		Weighted mode	17		0.80	0.62–1.03	0.10	
		Simple mode	17		0.79	0.61–1.03	0.11	
2	GCST90200190	IVW	17	>10	0.84	0.76–0.93	0.00	0.86
		MR-Egger	17		0.73	0.57–0.95	0.03	
		Weighted median	17		0.83	0.73–0.96	0.01	
		Weighted mode	17		0.84	0.68–1.03	0.11	
		Simple mode	17		0.82	0.65–1.02	0.10	
3	GCST90200767	IVW	17	>10	0.85	0.77–0.94	0.00	0.56
		MR-Egger	17		0.77	0.59–1.00	0.07	
		Weighted median	17		0.83	0.72–0.96	0.01	
		Weighted mode	17		0.83	0.68–1.00	0.07	
		Simple mode	17		0.84	0.68–1.03	0.10	
4	GCST90199704	IVW	17	>10	0.84	0.76–0.94	0.00	0.58
		MR-Egger	17		0.76	0.58–1.00	0.07	
		Weighted median	17		0.85	0.74–0.99	0.03	
		Weighted mode	17		0.78	0.62–0.98	0.05	
		Simple mode	17		0.73	0.56–0.95	0.03	
5	GCST90200378	IVW	17	>10	1.17	1.06–1.30	0.00	0.62
		MR-Egger	17		1.21	0.93–1.58	0.18	
		Weighted median	17		1.12	0.98–1.29	0.11	
		Weighted mode	17		1.09	0.88–1.36	0.43	
		Simple mode	17		1.05	0.82–1.35	0.69	
6	GCST90200367	IVW	17	>10	0.85	0.77–0.94	0.00	0.60
		MR-Egger	17		0.67	0.52–0.88	0.01	
		Weighted median	17		0.86	0.75–0.99	0.04	
		Weighted mode	17		0.87	0.69–1.10	0.27	
		Simple mode	17		0.89	0.68–1.15	0.37	
7	GCST90200167	IVW	17	>10	0.86	0.77–0.95	0.00	0.65
		MR-Egger	17		0.74	0.57–0.97	0.04	
		Weighted median	17		0.86	0.75–1.00	0.04	
		Weighted mode	17		0.92	0.72–1.18	0.52	
		Simple mode	17		0.91	0.71–1.18	0.51	
8	GCST90200173	IVW	17	>10	0.86	0.78–0.95	0.00	0.54
		MR-Egger	17		0.83	0.64–1.08	0.17	
		Weighted median	17		0.84	0.74–0.97	0.02	
		Weighted mode	17		0.81	0.64–1.02	0.09	
		Simple mode	17		0.80	0.62–1.03	0.11	
9	GCST90200249	IVW	17	>10	0.86	0.78–0.95	0.00	0.45
		MR-Egger	17		0.74	0.57–0.96	0.04	
		Weighted median	17		0.82	0.71–0.94	0.01	
		Weighted mode	17		0.81	0.65–1.02	0.09	
		Simple mode	17		0.64	0.64–1.03	0.10	
10	GCST90200393	IVW	17	>10	0.86	0.77–0.95	0.00	0.37
		MR-Egger	17		0.77	0.59–1.02	0.09	
		Weighted median	17		0.86	0.75–1.00	0.04	
		Weighted mode	17		0.86	0.69–1.07	0.19	
		Simple mode	17		0.90	0.72–1.13	0.39	

### C. Untargeted metabolomic characteristics of sepsis patients

**FIG. 5.**
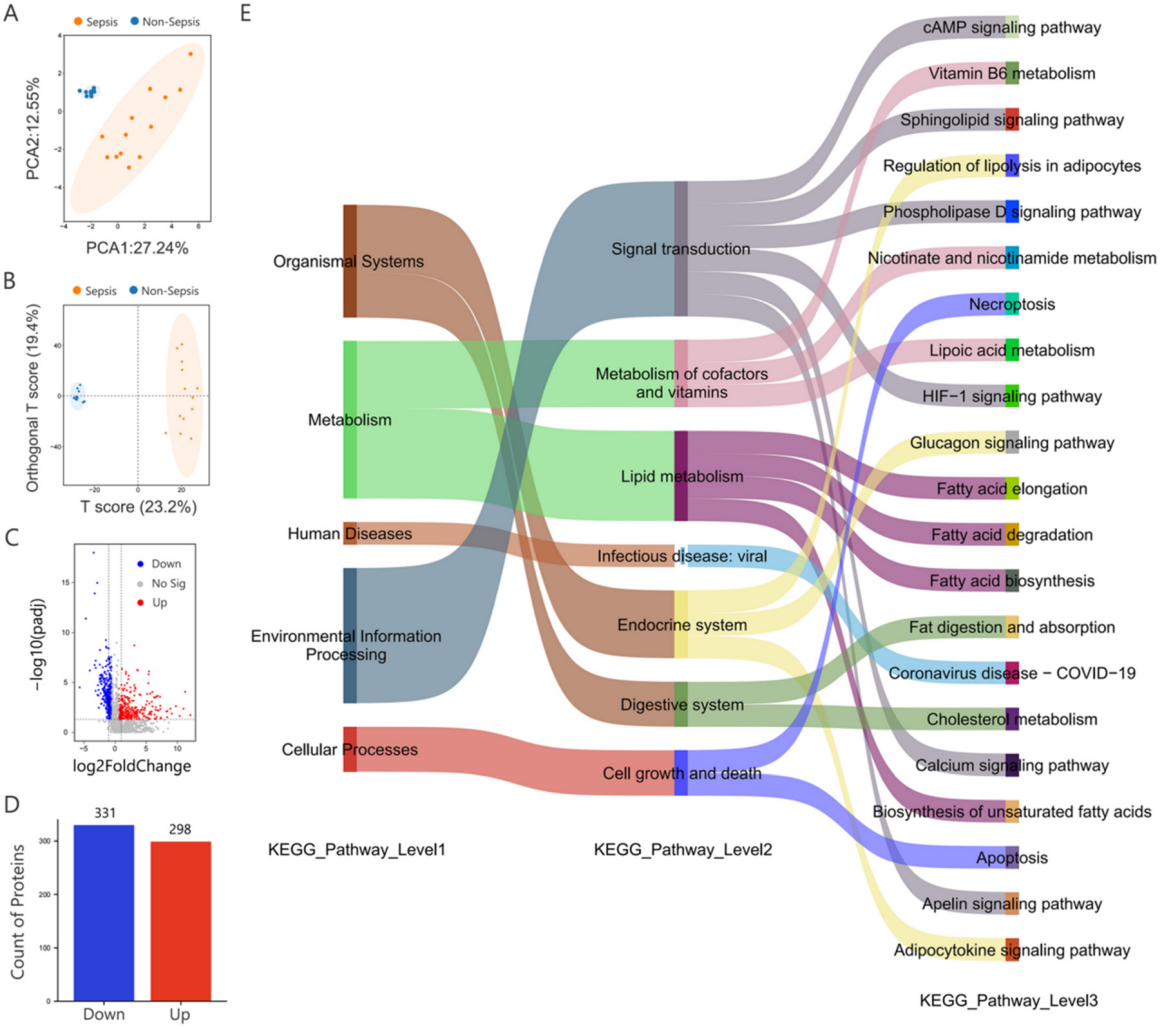
Untargeted metabolomics analysis in sepsis patients. (a) PCA plot showing the distribution of samples from the two groups. (b) PLS-DA plots depicting metabolite features under positive and negative ionization modes. (c) Volcano plot illustrating differential metabolites between the two groups. (d) Bar chart illustrating the regulatory changes in differential metabolites in sepsis. (e) Sankey diagram highlighting the metabolic pathways impacted by differential metabolites in sepsis.

Blood samples were collected from 13 sepsis patients (9 males, 4 females; mean age 57.92 ± 2.85 years) and 10 non-sepsis donors (6 males, 4 females; mean age 59.22 ± 2.14 years). Among the sepsis patients, three (23.08%) had cardiovascular disease, two (15.38%) had nephrosis, and six (46.15%) had diabetes. Among the non-sepsis donors, one (10.00%) had cardiovascular disease, one (10.00%) had nephrosis, and five (50.00%) had diabetes. There were no statistically significant differences between the two groups in terms of gender, age, or comorbidities (*P* > 0.05, Table III). Untargeted metabolomic analysis was performed on serum samples using LC–MS technology to identify significant differences in metabolite profiles between the sepsis and non-sepsis groups. Principal component analysis (PCA) was conducted to evaluate the clustering of samples within each group and their overall distribution trends. The PCA results demonstrated distinct clustering of serum samples from the sepsis and non-sepsis groups, indicating strong intra-group consistency and significant inter-group differences [Fig. 5(a)]. To further validate these differences, a supervised partial least squares discriminant analysis (PLS-DA) model was constructed using metabolite data from both positive and negative ion modes. The PLS-DA results effectively distinguished the metabolic profiles of the sepsis and non-sepsis groups under both ionization modes [Fig. 5(b)]. PERMANOVA analysis confirmed significant differences in metabolite profiles between sepsis and non-sepsis groups (supplementary material Fig. S3). The Bray–Curtis dissimilarity matrix showed high inter-group dissimilarity values, further supporting the significant separation between groups (supplementary material Table S6). A volcano plot [Fig. 5(c)] identified 629 differentially expressed metabolites between the two groups, including acetate, palmitic acid, butyric acid, and isovaleric acid. Of these, 298 metabolites were upregulated in the sepsis group, while 331 were downregulated. Hierarchical clustering of the differentially expressed metabolites further emphasized the metabolic distinctions between the serum samples of the sepsis and non-sepsis groups [Fig. 5(d)].

**TABLE III.** The baseline data of the two groups.

Characteristics	Sepsis group	Non-sepsis group	Χ^2^/t	*P*
Sex			0.212	0.685
Male	9 (69.23%)	6 (60.00%)		
Female	4 (30.77%)	4 (40.00%)		
Age (x ± s, year)	57.92 ± 2.85	59.22 ± 2.14	0.115	0.738
Underlie disease				
Cardiovascular disease	3 (23.08%)	1 (10.00%)	0.673	0.604
Nephrosis	2 (15.38%)	1 (10.00%)	0.144	0.602
Diabetes	6 (46.15%)	5 (50.00%)	0.034	0.593

The identified differential metabolites were analyzed using the KEGG database to elucidate the pathways through which they influence the host. The results [Fig. 5(e)] revealed that these metabolites were primarily involved in pathways related to metabolism, organismal systems, human diseases, environmental information processing, and cellular processes. Within the metabolism category, the most significant pathways were related to lipid metabolism, including fatty acid biosynthesis, fatty acid elongation, fatty acid degradation, and biosynthesis of unsaturated fatty acids. Additional pathways were identified in the metabolism of cofactors and vitamins, such as lipoic acid metabolism, nicotinate and nicotinamide metabolism, and vitamin B6 metabolism. In the organismal systems category, pathways were primarily linked to the digestive system (e.g., fat digestion and absorption, cholesterol metabolism) and the endocrine system (e.g., regulation of lipolysis in adipocytes, glucagon signaling pathway). Notably, within the cellular processes category, significant pathways were associated with cell growth and death, specifically apoptosis and necroptosis. These cell death pathways are critical for the formation of the PANoptosome complex, a key structure in programmed cell death known as PANoptosis, which plays a pivotal role in the pathogenesis of sepsis.

By integrating the MR analysis results (which identified *Prevotella 9* and fatty acid-related metabolites as causally associated) with the untargeted metabolomic analysis of clinical samples, it was observed that fatty acids not only exhibited a significant causal association with *Prevotella 9* but also demonstrated substantial differences between sepsis patients and non-sepsis subjects. These findings suggest that fatty acids may play a critical role in the pathophysiology of sepsis and hold potential as therapeutic targets or biomarkers.

### D. Identification and functional analysis of PFPGs in sepsis

**FIG. 6.**
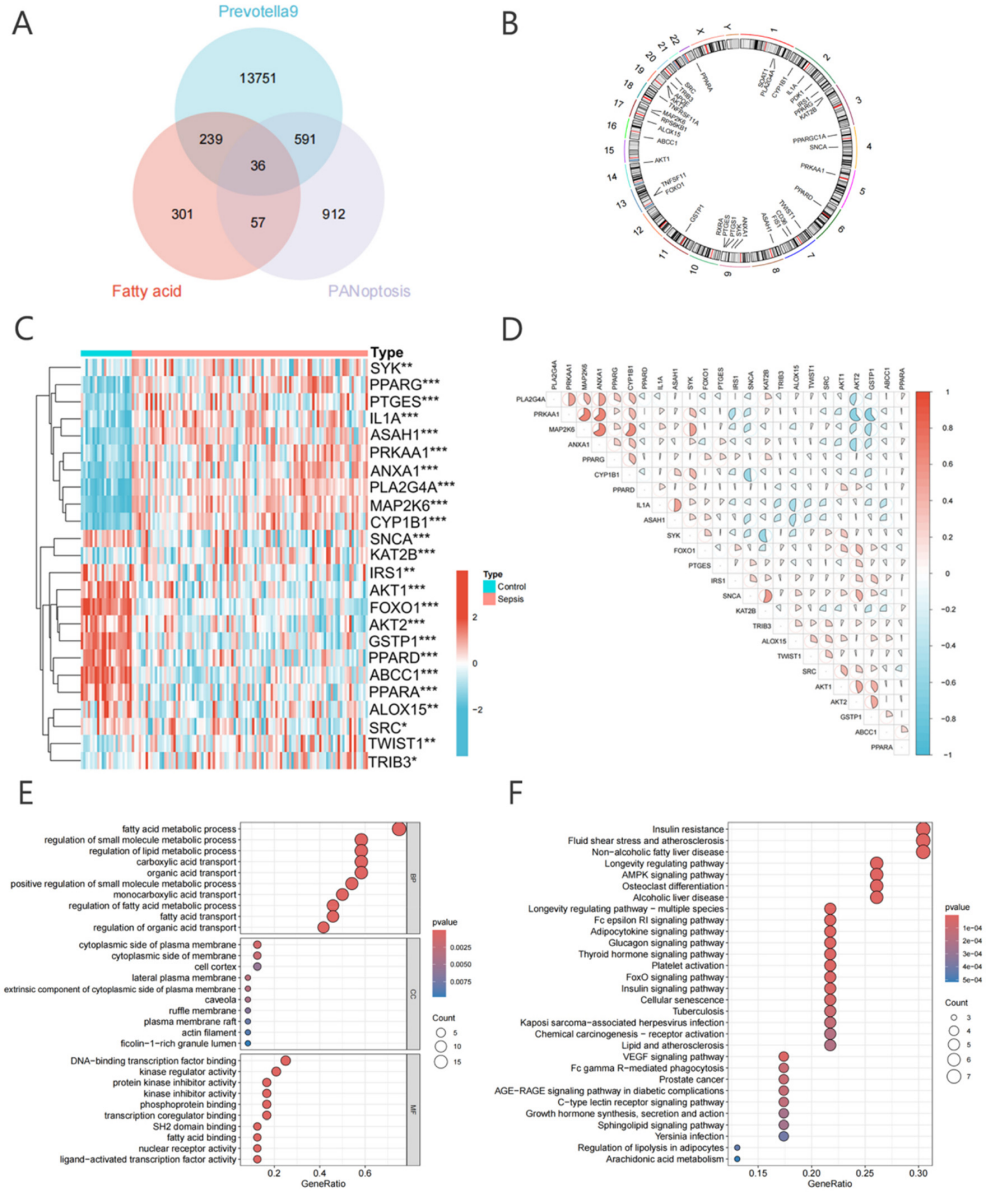
Genetic topology of PFPGs in sepsis. (a) Venn diagram highlighting the shared genes between the *Prevotella 9*, fatty acids, and PANoptosis-related genes. (b) CNV alteration sites of PFPGs, illustrating genetic variations associated with sepsis. (c) Expression levels of 24 differentially expressed PFPGs compared between normal and sepsis samples. (d) Correlation heatmap showing the relationships among differentially expressed PFPGs. (e) and (f) Enrichment analyses of differentially expressed PFPGs.

A total of 36 intersecting genes related to *Prevotella 9*, fatty acids, and PANoptosis were identified, collectively referred to as PFPGs [Fig. 6(a)]. Expression data for these PFPGs were extracted from all samples, and using the “limma” package, 24 differentially expressed PFPGs were identified. Among these, 11 differentially expressed PFPGs were significantly downregulated, while 13 were significantly upregulated (supplementary material Table S3). The chromosomal localization of the differentially expressed PFPGs was visualized using a Circos plot [Fig. 6(b)]. Further analysis indicated that the majority of upregulated differentially expressed PFPGs were predominantly observed in sepsis samples. These differentially expressed PFPGs exhibited primarily positive correlations in their expression patterns [Figs. 6(c) and 6(d)]. To explore the biological functions of the 24 differentially expressed PFPGs, enrichment analyses were performed using the Gene Ontology (GO) and KEGG databases. The results revealed that the differentially expressed PFPGs were involved in key biological processes, including metabolism and transcription. They were significantly enriched in several pathways related to metabolism, inflammation, and immunity, including the AMPK signaling pathway, Fc epsilon RI signaling pathway, and adipocytokine signaling pathway [Figs. 6(e) and 6(f)].

### E. Identification of key diagnostic genes for sepsis

**FIG. 7.**
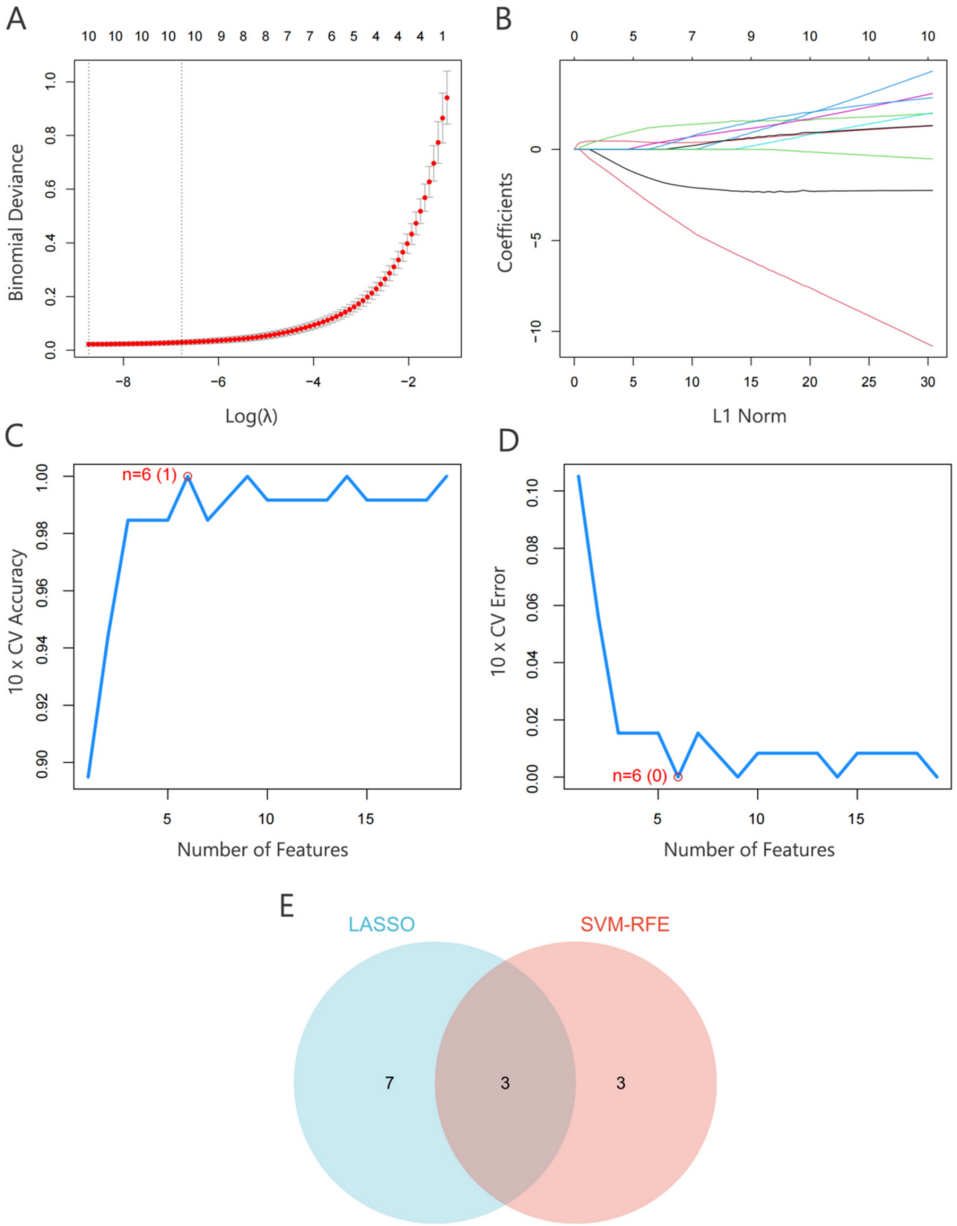
Identification of PFPG-related diagnostic genes in sepsis. (a) Number of feature genes identified using LASSO analysis. (b) LASSO coefficient curves for the selected feature genes. (c) and (d) SVM-Accuracy and SVM-Error plots showing the performance of the SVM-RFE algorithm. (e) Venn diagram highlighting the key diagnostic genes identified through the intersection of LASSO and SVM-RFE machine learning algorithms. LASSO: Least Absolute Shrinkage and Selection Operator; SVM-RFE: Support Vector Machine Recursive Feature Elimination.

To identify key diagnostic genes for sepsis, Least Absolute Shrinkage and Selection Operator (LASSO) analysis and Support Vector Machine Recursive Feature Elimination (SVM-RFE) machine learning methods were employed. LASSO analysis, a widely used approach for predictive modeling, utilizes cross-validation to determine the hyperparameter lambda (λ), effectively minimizing the risk of overfitting. The number of feature genes selected through LASSO analysis and the corresponding LASSO coefficient curve are shown in Figs. 7(a) and 7(b). Using this method, ten PFPG-related feature genes associated with sepsis (*ABCC1*, *PLA2G4A*, *MAP2K6*, *TRIB3*, *IL1A*, *PPARG*, *ASAH1*, *FOXO1*, *GSTP1*, and *CYP1B1*) were identified for further analysis. In parallel, the SVM-RFE machine learning algorithm was applied to identify PFPG-related feature genes associated with sepsis. The results demonstrated that the minimum error rate, corresponding to the highest classification accuracy, was achieved when the number of features was reduced to six (*CYP1B1*, *ABCC1*, *PPARG*, *PPARA*, *TWIST1*, and *ANXA1*), as illustrated in Figs. 7(c) and 7(d). By integrating the results from both LASSO analysis and SVM-RFE, three overlapping feature genes (*ABCC1*, *CYP1B1*, and *PPARG*) were identified as key diagnostic genes for sepsis [Fig. 7(e)]. These genes may provide valuable insights into the molecular mechanisms of sepsis and hold potential as biomarkers for early diagnosis and therapeutic intervention.

### F. Performance validation of key diagnostic genes for sepsis

**FIG. 8.**
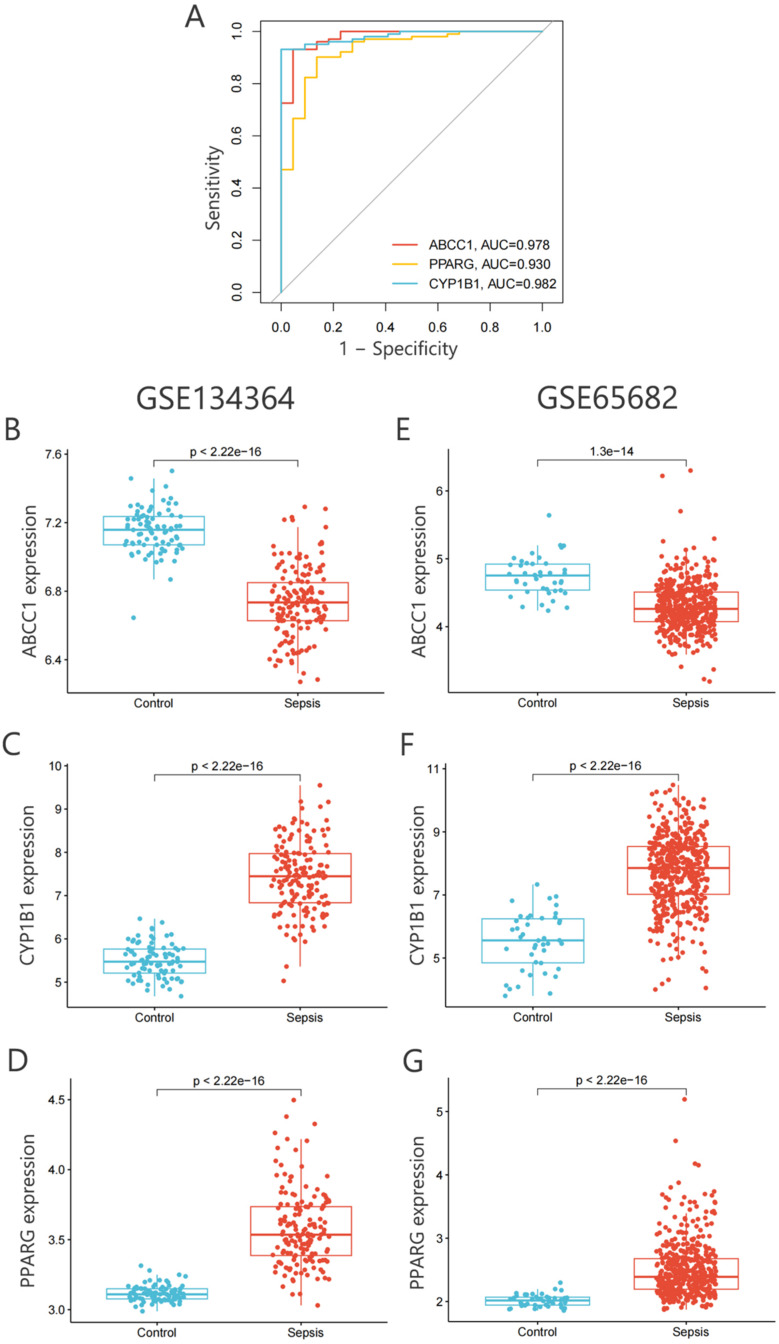
Diagnostic value of key diagnostic genes. (a) ROC curves illustrating the diagnostic performance of the three key diagnostic genes. (b)–(d) Expression levels of *ABCC1*, *CYP1B1*, and *PPARG* in dataset GSE134364. (e)–(g) Expression levels of *ABCC1*, *CYP1B1*, and *PPARG* in dataset GSE65682. ROC: receiver operating characteristic curve; AUC: area under the ROC curve.

The receiver operating characteristic (ROC) curve, a widely used method for evaluating diagnostic accuracy, plots sensitivity on the y axis and specificity on the x axis, offering an intuitive assessment of diagnostic performance across various thresholds. The area under the ROC curve (AUC) serves as a quantitative measure of performance, ranging from 0.5 to 1.0. An AUC closer to 1.0 indicates excellent diagnostic ability, whereas an AUC closer to 0.5 reflects poor performance. ROC curve analysis was performed to evaluate the diagnostic value of *ABCC1*, *CYP1B1*, and *PPARG* in sepsis patients. As shown in Fig. 8(a), all three key diagnostic genes demonstrated exceptional diagnostic performance, with AUC values of *ABCC1* (0.978), *CYP1B1* (0.982), and *PPARG* (0.930). Each gene achieved an AUC greater than 0.7 and close to 1.0, indicating strong diagnostic capability. Notably, the combined diagnostic model of these three genes achieved a perfect AUC of 1.000, highlighting their outstanding potential for accurately identifying sepsis patients (supplementary material Fig. S2). Further validation of these diagnostic genes was conducted using two independent sepsis datasets (*GSE134364* and *GSE65682*). The results confirmed the diagnostic significance of *ABCC1* [Figs. 8(b) and 8(e)], *CYP1B1* [Figs. 8(c) and 8(f)], and *PPARG* [Figs. 8(d) and 8(g)]. Among sepsis patients, *ABCC1* was identified as a low-expression gene, while *CYP1B1* and *PPARG* were identified as high-expression genes. These findings demonstrate that the three key diagnostic genes identified in this study (*ABCC1*, *CYP1B1*, and *PPARG*) are robust and broadly applicable, providing reliable biomarkers for the diagnosis of sepsis.

### G. Validation of key diagnostic genes for sepsis

**FIG. 9.**
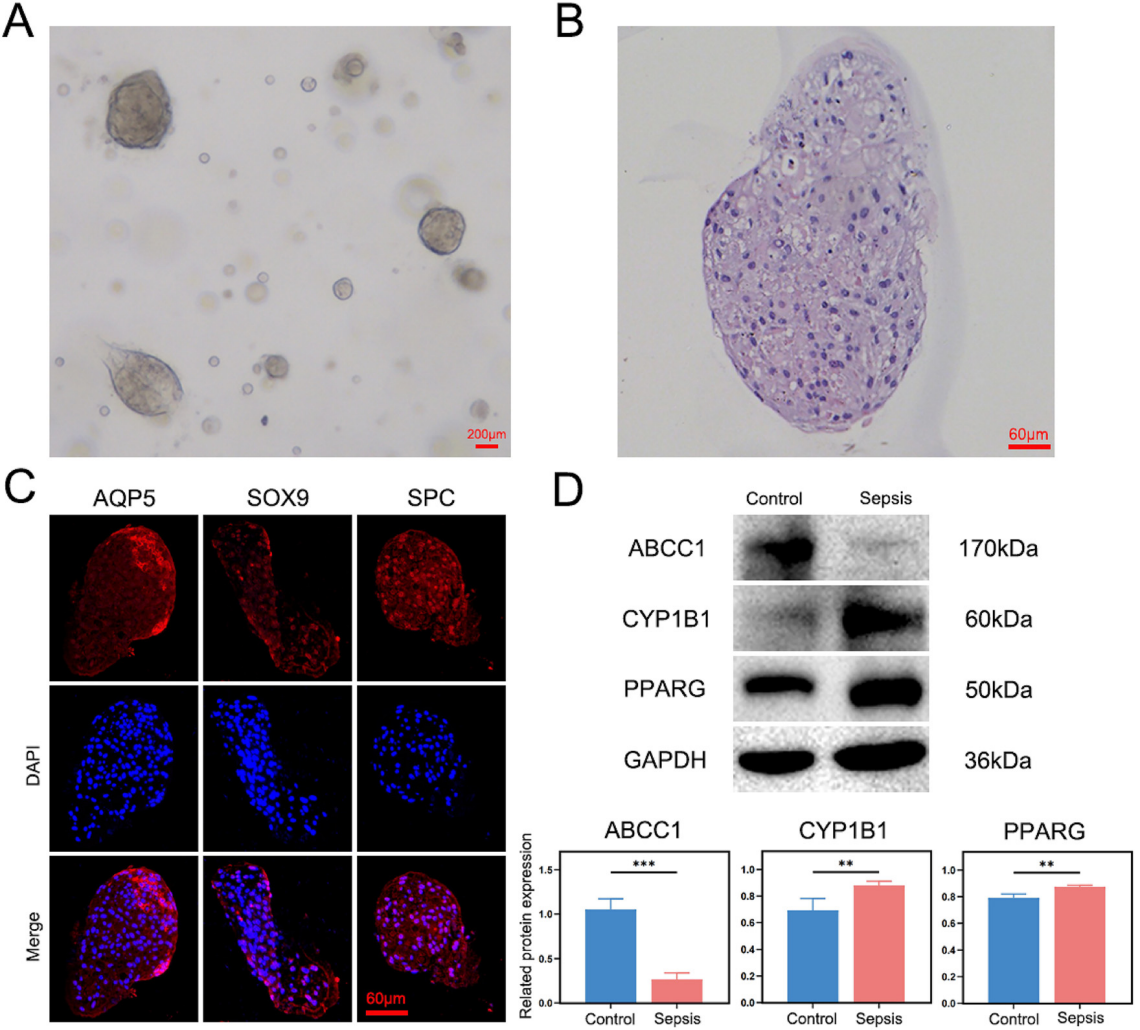
Expression of key diagnostic genes in human alveolar organoids derived from pulmonary sepsis patients. (a) Representative bright-field image of human alveolar organoids cultured for 7 days; scale bar = 200 *μ*m. (b) HE staining of human alveolar organoids. (c) Immunofluorescence quantification of AQP5, SOX9, and SPC in human alveolar organoids. (d) WB analysis showing the expression levels of ABCC1, CYP1B1, and PPARG in human alveolar organoids.

Organoid technology has emerged as a powerful tool for simulating disease models, replicating the structural, biological, and functional characteristics of organs.^14^ To validate the diagnostic applicability of the identified key genes for sepsis, human alveolar organoids were developed from lung-origin sepsis patients to simulate an *in vivo* disease model [Figs. 9(a) and 9(b)]. Immunofluorescence quantification was performed to assess the expression of aquaporin 5 (AQP5), transcription factor SOX9, and surfactant-associated protein C (SPC) in the disease-state human alveolar organoids. The results demonstrated high-expression levels of the alveolar epithelial marker AQP5 and the alveolar type II (AT2) cell markers SOX9 and SPC, confirming the organoids' resemblance to alveolar tissue in a disease state [Fig. 9(c)]. To further evaluate the diagnostic potential of the identified key genes, Western blot (WB) analysis was conducted to examine the expression of ABCC1, CYP1B1, and PPARG in the human alveolar organoids derived from lung-origin sepsis patients. The analysis revealed that ABCC1 was expressed at low levels, while CYP1B1 and PPARG were expressed at high levels in these organoids [Fig. 9(d)]. These findings validate the diagnostic relevance of ABCC1, CYP1B1, and PPARG in a simulated disease model, further supporting their potential as biomarkers for sepsis diagnosis.

**FIG. 10.**
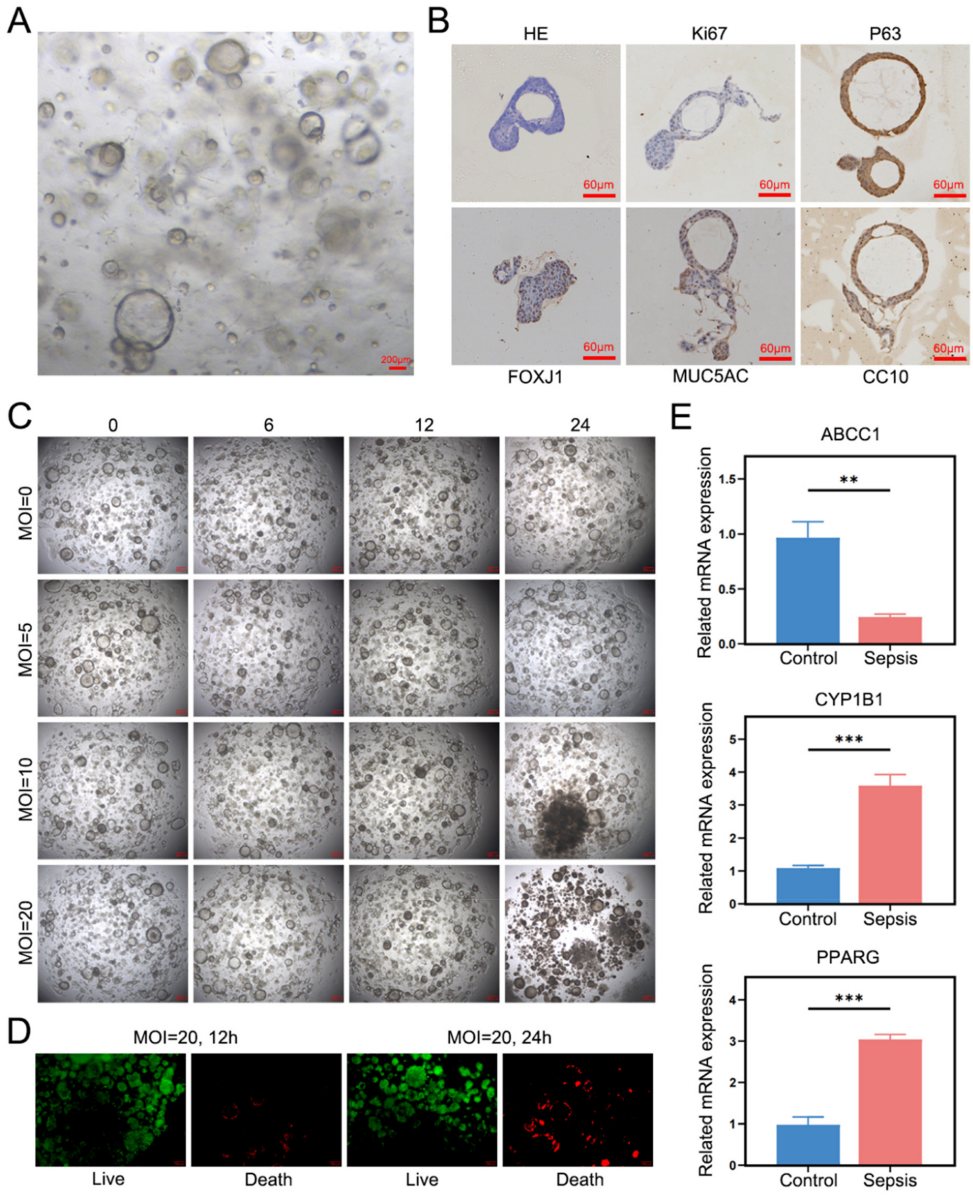
Expression of key diagnostic genes in bronchial organoids derived from pulmonary sepsis rats. (a) Representative bright-field image of rat bronchial organoids cultured for 7 days; scale bar = 200 *μ*m. (b) HE staining and IHC results for Ki67, P63, FOXJ1, MUC5AC, and CC10 in rat bronchial organoids. (c) Morphological status of rat bronchial organoids infected with four bacterial concentrations (MOI = 0, 5, 10, and 20) of *Escherichia coli* and *Staphylococcus aureus* at 0, 6, 12, and 24 h. (d) Fluorescence detection of organoid viability in rat bronchial organoids infected with MOI = 20 bacteria (*E. coli* and *S. aureus*) at 12 and 24 h. (e) RT-qPCR analysis quantifying the expression levels of *ABCC1*, *CYP1B1*, and *PPARG* in rat bronchial organoids.

To further validate the reliability of the identified key diagnostic genes, lung-origin sepsis rat bronchial organoids were constructed. Multiple cystic structures were observed [Fig. 10(a)], and hematoxylin–eosin (HE) staining revealed the presence of pseudostratified columnar ciliated epithelium and C-shaped hyaline cartilage. Immunohistochemical analysis confirmed the expression of key markers, including Ki67 (proliferating cell marker), P63 (basal cell marker), FOXJ1 (ciliated cell marker), MUC5AC (goblet cell marker), and CC10 (secretory cell marker) in the organoids [Fig. 10(b)]. These findings align with the characteristics of airway organoids, verifying the successful construction of rat bronchial organoids. To develop the lung-origin sepsis rat bronchial organoid model, bacterial solutions containing *E. coli* and *Staphylococcus* at concentrations of 0, 5, 10, and 20 MOI (multiplicity of infection) were used to infect the organoids. The survival rates of the organoids were monitored at 0, 6, 12, and 24 h. The results showed that as the MOI and incubation time increased, the survival rates of the organoids decreased, with the most significant reduction observed at an MOI of 20 [Fig. 10(c)]. Fluorescence analysis of organoids infected with MOI = 20 bacterial solution revealed that after 12 and 24 h of intervention, the number of dead organoids at 24 h was significantly higher compared to 12 h. These findings suggest that an MOI of 20 and a 24-h incubation period represent the optimal conditions for constructing a lung-origin sepsis rat bronchial organoid model [Fig. 10(d)]. Using rat bronchial organoids treated with MOI = 20 for 24 h, the expression of key diagnostic genes was validated. RT-qPCR analysis demonstrated that the expression patterns of the key diagnostic genes in the lung-origin sepsis rat bronchial organoids were consistent with those observed in human alveolar organoids derived from lung-origin sepsis patients. Specifically, *ABCC1* was downregulated, while *CYP1B1* and *PPARG* were upregulated in the rat bronchial organoids [Fig. 10(e)].

### H. GSEA, immune infiltration, and GSVA analyses of key diagnostic genes in sepsis

**FIG. 11.**
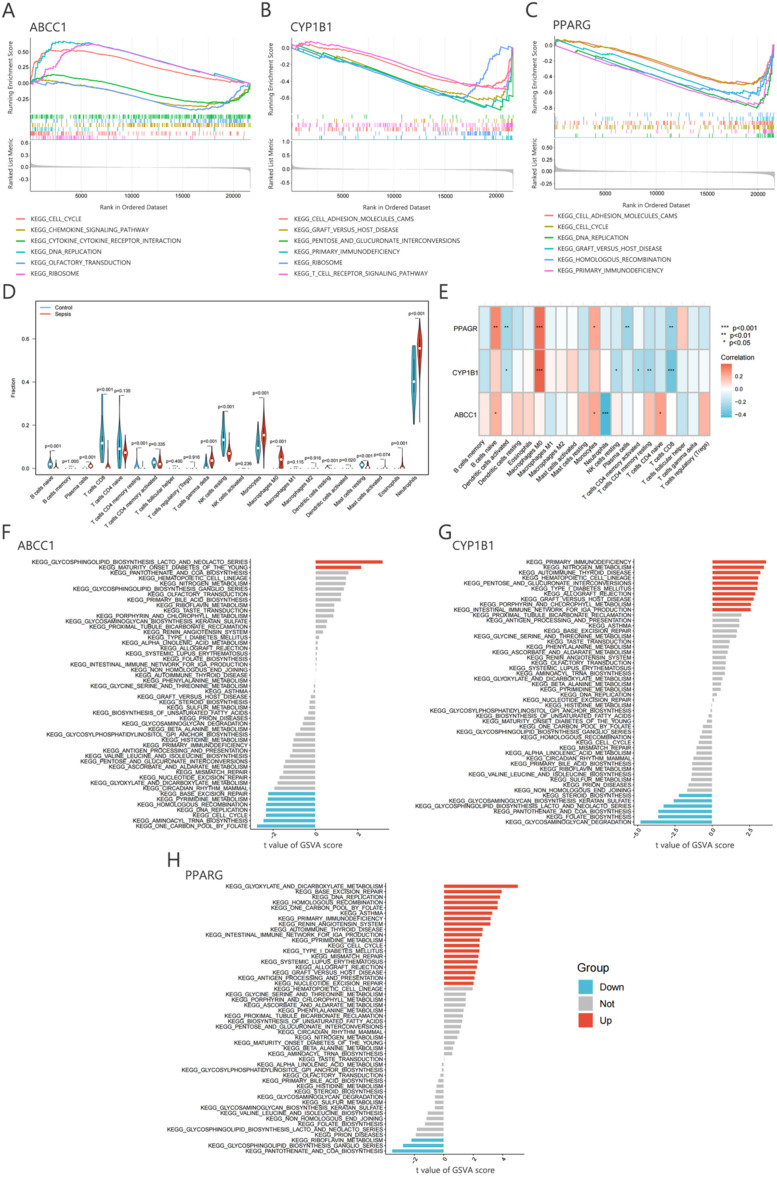
GSEA, immune infiltration, and GSVA analyses of key diagnostic genes. (a)–(c) GSEA analysis of *ABCC1*, *CYP1B1*, and *PPARG*, identifying enriched pathways associated with these genes. (d) Differences in immune infiltrating cell populations between healthy donors and sepsis patients. (e) Correlation analysis between *ABCC1*, *CYP1B1*, *PPARG*, and immune cells. (f) and (g) GSVA analysis of *ABCC1*, *CYP1B1*, and *PPARG.*

Gene Set Enrichment Analysis (GSEA) is a computational method used to identify biological pathways associated with specific gene sets, enabling the exploration of collective gene behavior in both healthy and diseased states.^15^ GSEA analysis of the three key diagnostic genes for sepsis (*ABCC1*, *CYP1B1*, and *PPARG*) revealed their involvement in immune processes mediated by cytokines, receptors, and kinases [Figs. 11(a)–11(c)]. To further investigate the relationship between these key diagnostic genes and immune cells, immune cell infiltration analysis was performed, focusing on 22 immune cell types, including T cells, B cells, and macrophages. Thirteen immune cell types exhibited significant differences in infiltration between healthy donors and sepsis patients. In sepsis patients, the infiltration of naive B cells, CD8 T cells, and resting NK cells was significantly reduced, whereas the infiltration of neutrophils, monocytes, and macrophages M0 was significantly elevated [Fig. 11(d)]. Correlation analysis demonstrated distinct relationships between the key diagnostic genes and immune cell infiltration. *ABCC1* was positively correlated with naive B cells and naive CD4 T cells, but negatively correlated with neutrophils. *CYP1B1* was significantly associated with six immune cell types, showing a strong positive correlation with macrophages M0. *PPARG* was positively correlated with naive B cells, macrophages M0, and monocytes, while negatively correlated with T cells CD8, plasma cells, and dendritic cells activated [Fig. 11(e)]. To further explore the regulatory roles of the key diagnostic genes in sepsis, Gene Set Variation Analysis (GSVA) was conducted. The results showed that *ABCC1* exerted both positive and negative regulatory effects on metabolic pathways [Fig. 11(f)]. Sixteen pathways were differentially regulated between high and low *CYP1B1* expression levels. *CYP1B1* upregulated pathways such as primary immunodeficiency, nitrogen metabolism, porphyrin and chlorophyll metabolism, and intestinal immune network for IgA production, while downregulating pathways like pantothenate and CoA biosynthesis and folate biosynthesis. These findings suggest that *CYP1B1* primarily functions through immune and metabolic pathways [Fig. 11(g)]. Similarly, *PPARG* was found to regulate biological functions in sepsis patients via immune and metabolic pathways [Fig. 11(h)].

### I. Single-cell analysis

**FIG. 12.**
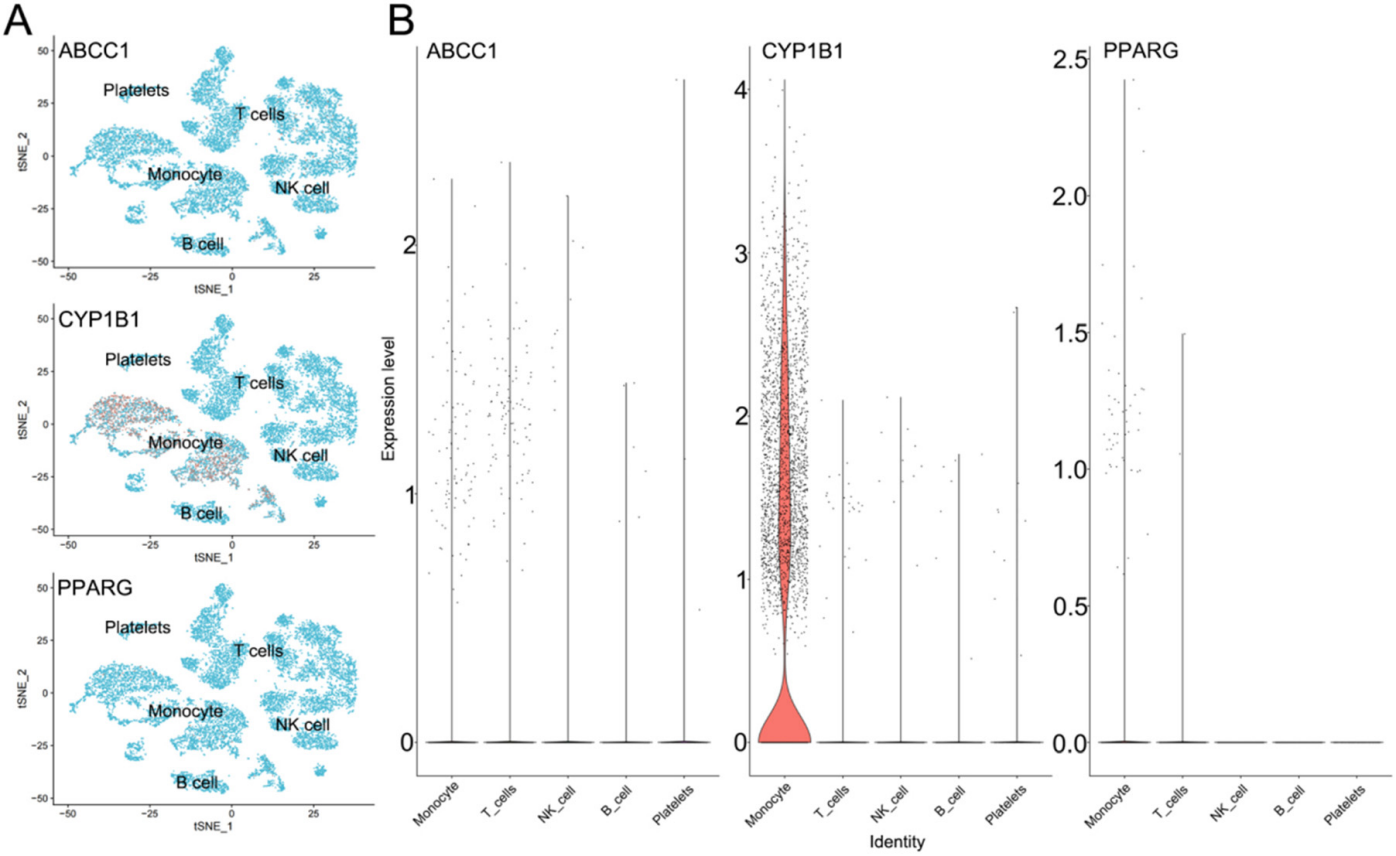
Single-cell analysis of *ABCC1*, *CYP1B1*, and *PPARG*. (a) and (b) Expression of *ABCC1*, *CYP1B1*, and *PPARG* in different immune cells.

Single-cell data from PBMC (peripheral blood mononuclear cell) samples of four sepsis patients were analyzed using several R packages, including “limma,” “Seurat,” “dplyr,” “magrittr,” “celldex,” “SingleR,” and “monocle.” Initial filtering of the single-cell data was performed with thresholds of logFC = 1 and adj-*P*-value = 0.05, ensuring the identification of statistically significant gene expression. The filtered data were normalized and subjected to dimensionality reduction analysis. Using t-SNE clustering and cell annotation, five distinct cell groups were identified: monocytes, T cells, NK cells, B cells, and platelets. To further investigate the roles of the key diagnostic genes (*ABCC1*, *CYP1B1*, and *PPARG*), which are associated with *Prevotella 9*, fatty acids, and PANoptosis, their expression profiles were examined across these five cell groups. The analysis revealed the following trends: *CYP1B1* expression was elevated across all five cell groups, with particularly high levels observed in monocytes. *ABCC1* and *PPARG* also exhibited increased expression, with a notable trend of upregulation in monocytes (Fig. 12). These findings suggest that the key diagnostic genes identified in this study may contribute to the onset and progression of sepsis by modulating monocyte activity.

## III. DISCUSSION

This study systematically uncovered the causal mechanisms and key molecular targets by which gut microbiota and their associated metabolites influence sepsis, integrating MR and multi-omics analyses. The MR analysis identified causal relationships between seven gut microbiota and sepsis. Among these, *Butyricicoccus*, *Prevotella 9*, *Ruminococcaceae UCG014*, and *Firmicutes* were negatively associated with sepsis, suggesting their protective roles. Conversely, *Collinsella*, *Ruminococcus 2*, and *Odoribacter* were positively associated with sepsis, highlighting their roles as risk factors in its development. Notably, the protective role of *Prevotella 9* was strongly supported by multiple MR evaluation methods, including IVW, Weighted Median, and Simple Mode. A prospective study^16^ further corroborated this finding, indicating that an increased abundance of *Prevotella 9* in the gut microbiota is associated with improved health outcomes. The study also emphasized the significant role of lipid metabolism in individual health. Additional evidence supporting the protective role of *Prevotella 9* comes from a recent study by Zhao *et al.*, who constructed a sepsis rat model. Their results demonstrated that modulation of the gut microbiota and their metabolites alleviated sepsis-related neurological damage. In this model, an increased abundance of *Prevotella 9* was observed, accompanied by the production of short-chain fatty acids (SCFAs) that contributed to maintaining the integrity of the gut barrier.^17^

Using MR analysis, 122 metabolites were identified as being causally associated with *Prevotella 9*. By integrating MR with untargeted metabolomic analyses, we found that fatty acids and their derivatives involved in lipid metabolism (e.g., acetate, palmitic acid, butyric acid, and isovaleric acid) are associated with the prognosis of sepsis patients. Fatty acids are essential biomolecules that participate in complex metabolic pathways, exerting significant biological effects on human health.^18^ Recent studies^19^ have further validated their critical role in the pathophysiology of sepsis. In this study, we identified acetate, palmitic acid, butyric acid, and isovaleric acid as sepsis-associated metabolites. Notably, *Prevotella 9* was negatively correlated with both branched- and straight-chain fatty acids (e.g., palmitic acid and isovaleric acid). Acetate and butyric acid, classified as short-chain fatty acids (SCFAs), exhibit diverse regulatory functions. Sepsis patients experience severe systemic inflammatory responses caused by pathogenic infections, coupled with immunosuppression that impairs immune function.^20^ SCFAs, such as acetate and butyric acid, have been shown to exert immunomodulatory and anti-inflammatory effects.^21^ Specifically, they inhibit M2 macrophage polarization in an FFAR2-dependent manner, thereby mitigating inflammation.^22^ Moreover, multiple studies have demonstrated that SCFAs, particularly acetate and butyric acid, alleviate sepsis-associated encephalopathy by regulating neuroinflammation, immunity, and energy metabolism.^23–25^ On the other hand, palmitic acid has been shown to exert detrimental effects. Choroszy *et al.*^26^ demonstrated that palmitic acid exacerbates the toxic effects of metabolic endotoxemia on vascular endothelium, indicating its harmful impact on the body. Similarly, Muratsu *et al.*^27^ investigated metabolite expression in a polymicrobial sepsis mouse model and reported reduced levels of metabolites such as butyric acid and isovaleric acid in the disease model. These findings align with the results of our study. Sepsis patients experience an excessive release of inflammatory mediators and dysregulated systemic inflammatory responses.^28^ The use of plasma metabolites in our study captures systemic metabolic alterations relevant to sepsis pathophysiology. However, cecal metabolites would provide more direct evidence of gut microbiota metabolic activity. Studies have shown that while some microbial metabolites (e.g., SCFAs) are readily absorbed and detectable in plasma, others remain localized in the gut. The fatty acids identified in our plasma analysis likely represent absorbed microbial products and host metabolic responses. Future studies incorporating both cecal and plasma metabolomics would provide a more comprehensive view of the gut–systemic metabolic axis in sepsis. In recent years, PANoptosis—a newly identified form of programmed cell death that integrates apoptosis, pyroptosis, and necroptosis—has been recognized as a significant contributor to the progression of sepsis. During sepsis, infection-induced inflammatory responses trigger PANoptosis in various cell types, including macrophages and neutrophils. While PANoptosis plays an essential role in modulating immune responses in sepsis patients, its excessive activation can lead to tissue damage and organ dysfunction.^29^ In our previous studies, we identified differential expression of PANoptosis-related pathways in sepsis models. Untargeted metabolomic analyses further revealed significant enrichment of apoptosis and necroptosis pathways, which are critical for the formation of PANoptosome complexes. These findings emphasize the pivotal role of PANoptosis in the development and progression of sepsis, underscoring its potential as a therapeutic target.

In summary, the development and progression of sepsis are closely linked to *Prevotella 9*, fatty acids, and PANoptosis. This study investigated the mechanisms underlying these associations from the perspective of PANoptosis and utilized machine learning algorithms to identify sepsis-specific diagnostic biomarkers. We identified 24 differentially expressed genes intersecting *Prevotella 9*, fatty acids, and PANoptosis (differentially expressed PFPGs) in sepsis. Functional enrichment analysis indicated that these differentially expressed PFPGs are primarily involved in pathways related to metabolism, inflammation, and immunity, including the AMPK signaling pathway, Fc epsilon RI signaling pathway, and adipocytokine signaling pathway. Using LASSO regression and SVM-RFE machine learning methods, we screened and identified three key diagnostic genes with significant diagnostic value: *ABCC1*, *CYP1B1*, and *PPARG*. Among these, *ABCC1* was found to be downregulated in sepsis, while *CYP1B1* and *PPARG* were upregulated. Validation using two independent sepsis datasets confirmed these consistent expression patterns. To further verify these findings, we constructed human alveolar organoids derived from lung-origin sepsis patients and bronchial organoids from lung-origin sepsis rat models. These organoid disease models, which replicate structural, biological, and functional characteristics,^14^ were used to examine the three diagnostic genes. The results demonstrated that *ABCC1* was significantly downregulated, while *CYP1B1* and *PPARG* were significantly upregulated in the sepsis organoids. These findings indicate that the three diagnostic genes identified in this study (*ABCC1*, *CYP1B1*, and *PPARG*) are reliable diagnostic biomarkers with broad applicability. Mitochondrial abnormalities in sepsis, including swelling, condensation, and disruption of the mitochondrial membrane, have been reported in multiple studies.^30,31^ These findings align with sepsis-related disruptions in cellular and metabolic homeostasis, underscoring the potential of mitochondrial dysfunction as a therapeutic target. *ABCC1* (multidrug resistance-associated protein 1), a member of the ATP-binding cassette (ABC) transporter superfamily,^32^ is closely associated with mitochondrial function. *ABCC1* maintains cellular homeostasis by exporting mitochondrial damage-associated molecular patterns (mito-DAMPs). Its downregulation may lead to the accumulation of mitochondrial DNA (mtDNA) in circulation, which activates the cGAS-STING pathway and exacerbates organ damage.^33,34^ Notably, studies have shown that *ABCC1* expression in mitochondria increases following drug treatment in disease models.^35^ The observed low expression of *ABCC1* in sepsis is strongly associated with mitochondrial damage mechanisms. The Wnt5A signaling pathway is activated in sepsis patients.^36^ Skaria *et al.*^37^ demonstrated that *CYP1B1* expression is upregulated in response to the pro-inflammatory mediator Wnt5A. Lipid-laden macrophages (LLMs) are frequently observed in inflammatory diseases and oxidative stress conditions. Zhu *et al.* showed that treatment of macrophages with the *CYP1B1* inhibitor TMS effectively inhibited CSE (cigarette smoke extract)-induced LLM formation.^38–40^ These findings suggest a strong association between *CYP1B1* overexpression, inflammation, and oxidative stress. *PPARG* plays a dual regulatory role in sepsis. It inhibits NF-κB to mitigate cytokine storms while suppressing fatty acid oxidation, which exacerbates immune paralysis.^41,42^ These findings suggest that *PPARG* may participate in metabolic reprogramming during different stages of sepsis.

GSEA and GSVA analyses of the three key diagnostic genes (*ABCC1*, *CYP1B1*, and *PPARG*) revealed their primary roles in regulating immune-related pathways and metabolic processes, aligning with the pathophysiological characteristics of sepsis, including immune hyperactivation and metabolic dysregulation.^43^ *ABCC1*, a transporter associated with chemokine secretion and neutrophil regulation, exhibited a significant negative correlation with neutrophil infiltration, suggesting its role in controlling excessive inflammation.^44,45^ *CYP1B1* is linked to oxidative stress and macrophage polarization.^40^ Our analysis showed a strong positive correlation between *CYP1B1* and M0 macrophages, implicating its involvement in driving the cytokine storm characteristic of sepsis. *PPARG*, a nuclear receptor regulating lipid metabolism and macrophage differentiation, demonstrated dual regulatory functions. It was positively correlated with monocytes but negatively correlated with CD8^+^ T cells, highlighting its potential role in modulating immune responses. Single-cell analysis further revealed that *ABCC1*, *CYP1B1*, and *PPARG* are highly expressed in monocytes, which are central to the immunopathology of sepsis, acting as key mediators of both inflammation and immune suppression.^46,47^ The significant upregulation of these genes in monocytes suggests their involvement in monocyte functional reprogramming. For instance, the association of *CYP1B1* with M0 macrophages supports its role in promoting a pro-inflammatory phenotype, potentially exacerbating tissue damage. Similarly, the dual regulatory effects of *PPARG* on metabolic pathways and immune cells underscore its role in metabolic adaptation during sepsis-induced immune suppression. In summary, these findings emphasize the pivotal role of monocytes in sepsis pathogenesis and suggest that they could serve as critical cellular targets for therapeutic intervention. Developing monocyte-targeted therapies may help mitigate immune dysfunction and improve clinical outcomes in sepsis.

Although this study has significantly advanced our understanding of the mechanisms underlying sepsis, several limitations should be acknowledged. First, while the use of a genetically randomized study design minimized the influence of confounding factors, the potential impact of unconsidered variables cannot be fully excluded. Further investigations are needed to validate the causal relationship between gut microbiota and sepsis. Second, our metabolomics analyses utilized circulating (plasma) metabolites rather than gut-derived (cecal) metabolites. While circulating metabolites reflect systemic metabolic status relevant to sepsis pathophysiology, they may not fully capture local gut microbiota–metabolite interactions. Cecal metabolites might provide more direct evidence of microbial metabolic activity. However, plasma metabolites offer the advantage of clinical accessibility and relevance to systemic inflammation in sepsis. Third, the integration of data from multiple clinical cohorts presents challenges, including potential heterogeneity in patient populations, disease severity, and treatment protocols. Although we used standardized analytical approaches and validated findings across independent datasets, residual heterogeneity may influence result interpretation. Future prospective studies with harmonized protocols would strengthen these findings. Fourth, the untargeted metabolomics validation cohort was relatively small (13 sepsis vs 10 controls). While we identified 629 significantly different metabolites with clear separation in PCA/PLS-DA analyses, suggesting robust differences, larger cohorts would enhance statistical power for detecting moderate effect sizes and strengthen generalizability. The consistency of our findings with the MR analysis of 12 301 sepsis patients provides additional confidence in our results. Fifth, although the three key diagnostic genes were preliminarily validated using organoid models, additional functional studies are necessary to elucidate their precise roles and mechanisms in sepsis pathogenesis. Finally, the sample size of this study was relatively limited. Validation of these findings in larger cohorts is essential to ensure their generalizability and robustness.

## IV. CONCLUSION

This study integrates Mendelian randomization and multi-omics analyses to systematically investigate the causal relationship between gut microbiota, their metabolites, and sepsis. Seven gut microbiota, encompassing both beneficial and harmful strains, were identified. Notably, *Prevotella 9* demonstrated potential protective effects against sepsis through its regulation of lipid metabolism. Furthermore, this study explored the mechanisms linking gut microbiota, their associated metabolites, and the inflammatory response in sepsis from the perspective of PANoptosis. Using machine learning algorithms, we identified three key diagnostic genes for sepsis (*ABCC1*, *CYP1B1*, and *PPARG*) with high reliability and broad applicability. These genes were shown to participate in immune–metabolic pathways, including neutrophil regulation, oxidative stress, and macrophage polarization, aligning with the pathophysiological characteristics of sepsis, such as immune hyperactivation and metabolic dysregulation. Additionally, the predominant expression of *ABCC1*, *CYP1B1*, and *PPARG* in monocytes underscores the critical role of monocyte functional reprogramming in sepsis pathogenesis. This highlights monocytes as promising cellular targets for sepsis interventions. In summary, this study provides novel insights into the immunometabolic dysregulation of the microbiome in sepsis, offering a strong foundation for the development of innovative diagnostic and therapeutic biomarkers to enhance sepsis management and improve patient outcomes.

## V. METHODS

### A. Study design

**FIG. 13.**
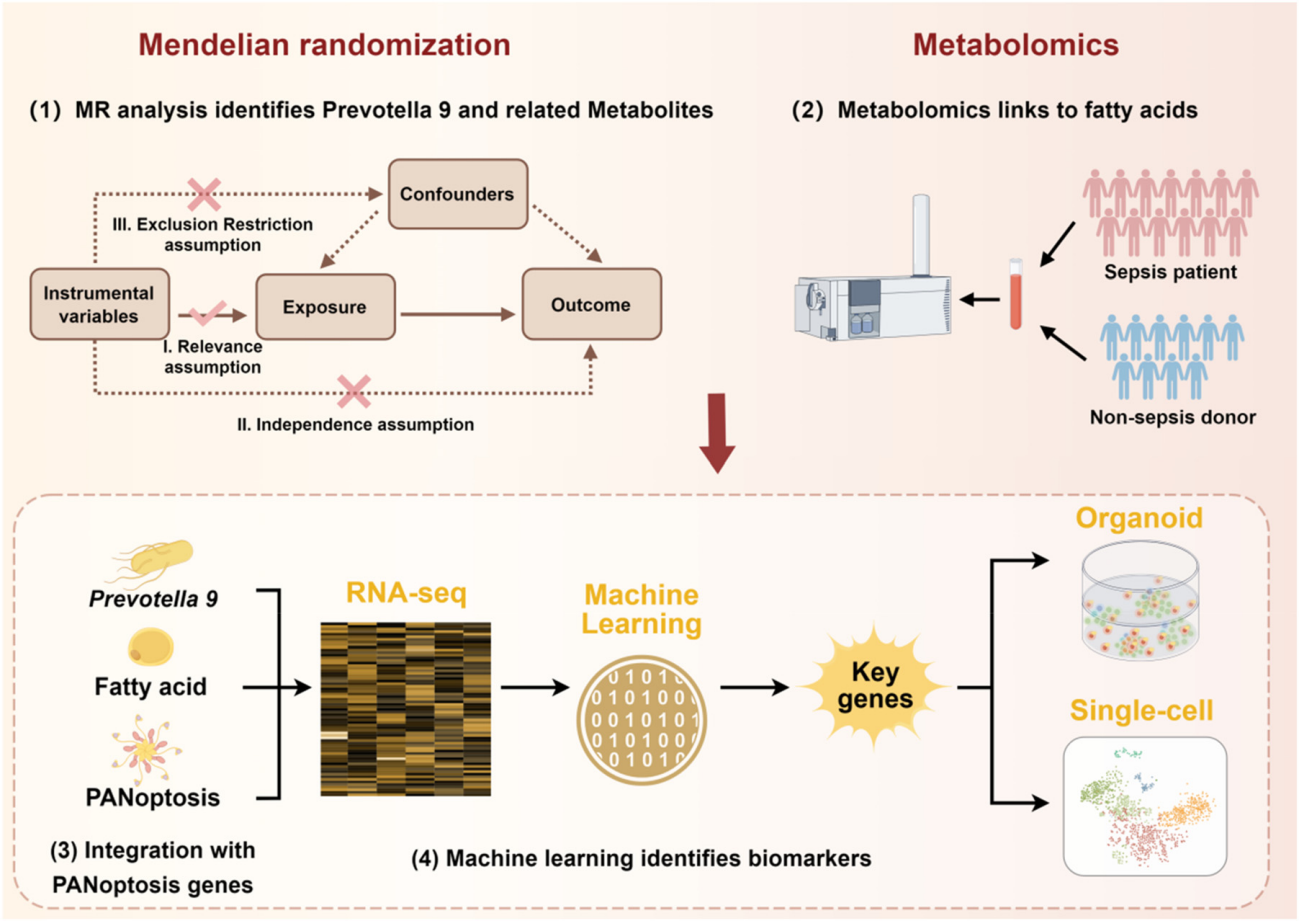
Research flow chart illustrating the integrated approach. The study begins with Mendelian randomization to identify causal gut microbiota (*Prevotella 9* and related metabolites), followed by metabolomics to discover fatty acid mediators. These findings are integrated with PANoptosis-related genes based on our previous work showing their dysregulation in sepsis. Machine learning algorithms identify diagnostic biomarkers from the intersection of these three domains, which are then validated in organoid models.

This study adhered to the STROBE-MR guidelines for Mendelian randomization (MR) epidemiological study designs,^48^ alongside the Strengthening the Reporting of Observational Studies in Epidemiology (STROBE) guidelines.^49^ These frameworks were instrumental in enhancing the quality, rigor, and standardization of the study protocol. The study design followed the three core assumptions of MR analysis: (I) Relevance: The instrumental variable (IV) used in this study demonstrated a significant association with the gut microbiota, specifically focusing on 119 genera with an average relative abundance >1% (excluding 12 unclassified genera). (II) Independence: The IV was associated with sepsis exclusively through its relationship with the gut microbiota and not through alternative pathways. (III). Exclusion restriction: The IV was free from associations with confounders directly linked to sepsis. Building on these MR principles, the study further investigated the metabolic pathways through which the gut microbiota influence sepsis. Untargeted metabolomics was employed to identify differential metabolites between sepsis patients and non-sepsis controls. Using bioinformatics and machine learning algorithms, key diagnostic genes for sepsis were identified and subsequently validated. Validation was conducted using lung-derived organoids developed from alveolar tissues of sepsis patients and bronchial organoids derived from sepsis-induced rat models. To explore the functional roles of these diagnostic genes, single-cell transcriptomic analyses and functional enrichment analyses were performed to elucidate their mechanisms of action. A detailed overview of the study design is presented in Fig. 13. The GWAS summary statistics and transcriptomic datasets of sepsis patients utilized in this study were publicly available, eliminating the need for separate ethical approval. While the GWAS summary statistics and transcriptomic datasets used in this study are publicly available and de-identified, our research protocol for secondary data analysis was reviewed by our institutional review board to ensure ethical compliance. The original data collection for these datasets was conducted under approved ethical protocols as documented in their respective publications.

### B. Data sources

The data utilized in this study were categorized into two primary components: (1) Mendelian randomization (MR) data: Sepsis GWAS dataset: Using “sepsis” as the keyword, a search was conducted within the FinnGen consortium's R9 release (https://r9.finngen.fi/). The selection of the dataset followed rigorous screening criteria: (I) datasets must contain confirmed sepsis diagnoses based on sepsis criteria; (II) minimum of 10 000 cases to ensure statistical power for MR analysis; (III) exclude puerperal sepsis and neonatal bacterial sepsis; and (IV) European ancestry to minimize population stratification. Among four candidate datasets identified, finngen_R9_AB1_OTHER_SEPSIS met all criteria with 12 301 sepsis cases and 332 343 controls. Gut microbiota genetic data: Genetic variation data for gut microbiota were obtained from the MiBioGen consortium (https://mibiogen.gcc.rug.nl/), which conducted a genome-wide meta-analysis of gut microbiota composition in 18 340 individuals (supplementary material Table S5).^50^ From the 131 identified genera with an average relative abundance >1%, 12 unclassified genera were excluded, leaving 119 known genera for analysis. Plasma metabolome data: Genomic data for plasma (circulating) metabolites were sourced from the study by Chen *et al.*,^51^ available through the GWAS summary statistics database (http://ftp.ebi.ac.uk/pub/databases/gwas/summary_statistics/). This dataset includes 1091 blood metabolites and 309 metabolite ratios (GCST90199621–GCST90201020). (2) Bioinformatics data: Transcriptomic data: RNA sequencing (RNA-seq) data were retrieved from the GEO database (https://www.ncbi.nlm.nih.gov/geo/) using “sepsis” as the keyword. For transcriptomic data selection, we applied the following criteria: (I) datasets must contain confirmed sepsis diagnoses based on sepsis criteria; (II) Affymetrix platforms; (III) gene expression data from whole blood; and (IV) availability of survival data. The dataset *GSE95233*, based on the GPL570 platform, was selected for its inclusion of 28-day survival information. It comprises RNA-seq data from 51 sepsis patients (GSM2500371–GSM2500472) and 22 healthy volunteers (GSM2500349–GSM2500370). GSE95233 was selected as the primary dataset meeting all criteria.^52^ The datasets *GSE65682* and *GSE134364*^53,54^ were utilized as validation datasets. Single-cell transcriptomic data: Single-cell transcriptomic data were obtained from *GSE151263*,^55^ which includes PBMC data from eight sepsis patients. However, data from four patients with acute respiratory distress syndrome (ARDS) were excluded, leaving data from four sepsis patients without ARDS for analysis. Results from untargeted metabolomics and SNP data (*P* < 0.05) associated with sepsis-related gut microbiota were integrated. These SNPs were converted into gene data using the g:Profiler online database (https://biit.cs.ut.ee/gprofiler/snpense), identifying 14 617 genes associated with the genus *Prevotella 9*. Using “fatty acid” as the keyword, a gene set of 633 fatty acid-related genes was retrieved from the Human Molecular Signatures Database (MSigDB, https://www.gsea-msigdb.org/gsea/msigdb/index.jsp).^56^ Additionally, 1596 PANoptosis-related genes were identified from the GeneCards database (https://www.genecards.org/) using a correlation score threshold of >3.^57^

### C. Untargeted metabolomics analysis of sepsis patients

Clinical data were collected from 13 sepsis patients treated at the First Affiliated Hospital of Guangxi University of Chinese Medicine between January 2024 and December 2024. These patients met the clinical diagnostic criteria for severe pulmonary infection and sepsis.^58,59^ Additionally, data were collected from ten non-sepsis donors. Participants in both groups were aged between 40 and 75 years, were permanent residents of Nanning, Guangxi, and had not undergone treatment with hormones, immunosuppressants, or organ transplantation within the past 6 months. Informed consent was obtained from all participants, who voluntarily provided baseline clinical information and blood samples. The study protocol adhered to the principles of the Declaration of Helsinki and was approved by the Independent Ethics Committee for the First Affiliated Hospital of Guangxi University of Chinese Medicine (Approval No. Lunshen2023-034-02).

Blood samples (5 ml of venous blood) were collected from all participants. Following pretreatment, the samples were analyzed using an Agilent 1290 Infinity ultrahigh-performance liquid chromatography (UHPLC) system, coupled with an Agilent 6545 UHD Accurate-Mass Q-TOF mass spectrometer, to perform untargeted metabolomics analysis.

### D. Mendelian randomization (MR) analysis

This study adhered to the fundamental assumptions of MR analysis in selecting instrumental variables. The specific selection criteria were as follows: (I) Significance threshold: Single-nucleotide polymorphisms (SNPs) with a significance level of *P* < 1 × 10^−5^ (1 × 10^−5^) were identified as potential instrumental variables. (II) Linkage disequilibrium (LD): SNPs with LD values of r^2^ > 0.001 were excluded. Since r^2^ values cannot be directly obtained from GWAS summary data, they were calculated using a standard formula in this study. (III) F-statistic: The F-statistic for each SNP was calculated. SNPs with F-statistics <10, considered weak instrumental variables, were excluded to minimize bias. Only SNPs with F-statistics >10 were included in subsequent analyses. After selecting appropriate SNPs, inverse variance weighting (IVW) was employed as the primary method for causal inference. To ensure robustness, complementary methods (MR-Egger, weighted median, simple mode, and weighted mode) were also applied. A leave-one-out sensitivity analysis was conducted by sequentially excluding each instrumental variable to assess whether any single SNP disproportionately influenced the causal estimates. MR-PRESSO (Mendelian Randomization Pleiotropy RESidual Sum and Outlier) analysis was performed to detect and correct for horizontal pleiotropy by identifying and removing significant outliers. The formula for calculating r^2^ in this study is as follows:

$$r^{2}=\sum \left[\frac{\left(\beta^{2}\cdot 2\cdot t\cdot (1-t)\right)}{\left(\beta^{2}\cdot 2\cdot t\cdot (1-t)+se^{2}\cdot 2\cdot y\cdot t\cdot (1-t)\right)}\right],$$

where *β* represents the effect size estimate for each SNP, *t* denotes the frequency of the effect allele, *y* represents the sample size, and *se* refers to the standard error of each SNP.

The identification of SNPs from the 119 microbiota genera followed this process: (I) The MiBioGen consortium performed 16S rRNA gene sequencing on fecal samples from 18 340 individuals; (II) Microbial abundance data were normalized and transformed to approximate normal distributions; (III) Genome-wide association analysis was performed, treating each genus abundance as a quantitative trait; (IV) SNPs showing significant associations (*P* < 1 × 10^−5^) with genus abundance were identified as potential instrumental variables; and (V) For each genus, we extracted SNPs meeting our IV selection criteria (F-statistic >10, r^2^ < 0.001). This resulted in 1232 genus-associated SNPs across the 119 genera, with each genus having between 9 and 18 qualifying SNPs serving as instrumental variables for the MR analysis.

All MR analyses were conducted using R software (version 4.3.2, R Foundation for Statistical Computing). The “TwoSampleMR” and “MRPRESSO” R packages were utilized, with IVW as the primary method supported by MR-Egger, weighted median, simple mode, and weighted mode analyses. The MR-PRESSO test was also employed to detect and adjust for horizontal pleiotropy. A significance threshold of *P* < 0.05 was used for all statistical analyses.

### E. Mapping SNPs to genes

The online database *g:SNPense* (https://biit.cs.ut.ee/gprofiler/snpense) was employed to map the human SNP rs-code list used in this study—specifically, SNP data closely associated with the genus *Prevotella 9* (the most robust protective association with sepsis across multiple MR methods)—to corresponding gene names. This tool was also used to predict the functional effects of the variants and identify genes matching the SNP data. The mapped genes formed the foundation for subsequent bioinformatics and machine learning analyses, which were focused on investigating potential biological pathways and developing predictive models relevant to sepsis.

### F. Identification of overlapping genes (PFPGs) among *Prevotella 9*, fatty acids, and PANoptosis

In prior studies conducted by our research group, a pulmonary sepsis model in rats was successfully established using lipopolysaccharide in combination with *S. aureus* and *E. coli*, following the methodology described by Wu *et al.*^60^ These studies demonstrated that key PANoptosis-related genes (*ZBP1*, *PIPK1*, and *PIPK3*) were abnormally expressed in the pulmonary sepsis model. Compared to the control group, rats in the pulmonary sepsis group exhibited significantly elevated expression levels of *ZBP1*, *PIPK1*, and *PIPK3* in lung tissues (supplementary material Fig. S1). To explore the combined effects of *Prevotella 9*, fatty acids, and PANoptosis on sepsis pathogenesis, we identified overlapping genes among these three factors, designated as PFPGs (*Prevotella 9*-Fatty acid-PANoptosis Genes). A Venn diagram, a commonly utilized tool for detecting and graphically representing shared genes among multiple datasets, was employed to facilitate this analysis.^61^

### G. Identification and functional analysis of differentially expressed PFPGs

To identify differentially expressed PFPGs distinguishing sepsis patients from healthy donors, expression data for PFPGs were extracted from the sepsis dataset (*GSE95233*) using R software and the “limma” package. Differentially expressed PFPGs were identified based on a significance threshold of *P* < 0.05. To further explore the functional roles and pathways associated with differentially expressed PFPGs, enrichment analyses were performed. These included GO and KEGG analyses, which were conducted using several complementary R packages.

### H. Selection of key genes based on LASSO analysis and SVM machine learning and performance validation

This study employed LASSO (Least Absolute Shrinkage and Selection Operator) analysis and SVM (Support Vector Machine) machine learning to identify key genes associated with sepsis progression. LASSO analysis was performed using the “glmnet” package to identify feature genes with significant discriminatory power between sepsis patients and healthy donors. A robust cross-validation process involving 1000 iterations ensured the stability and reliability of the key genes identified through LASSO analysis.^62^ To further minimize the risk of overfitting, SVM machine learning was applied alongside the Recursive Feature Elimination (RFE) algorithm to refine the selection of diagnostic feature genes for sepsis.^63^ Genes identified as overlapping between the LASSO and SVM analyses were designated as key diagnostic genes for sepsis. The diagnostic performance of these key genes was evaluated using receiver operating characteristic (ROC) curve analysis. Additionally, their expression levels were validated against external datasets (*GSE65682* and *GSE134364*) to assess their applicability and generalizability across diverse populations.

### I. GSEA, immune, and GSVA analyses of diagnostic key genes

Gene Set Enrichment Analysis (GSEA) is an unsupervised analytical method designed to evaluate the enrichment of predefined gene sets, focusing on broader biological contexts compared to GO enrichment analysis.^4^ Using GSEA,^64^ we investigated the biological mechanisms associated with the diagnostic key genes in sepsis. To further examine the role of these key genes, immune cell infiltration in sepsis patients and healthy donors was analyzed using the CIBERSORT algorithm. The relationship between key gene expression levels and immune cell infiltration was also explored. Additionally, the Gene Set Variation Analysis (GSVA) method was applied to evaluate changes in biological activity across sepsis patients with varying expression levels of the key genes. This analysis identified significantly regulated biological processes and provided insights into the functional states of these processes in the context of sepsis.

### J. Construction of human alveolar organoids for pulmonary sepsis

To model pulmonary sepsis *in vitro*, human alveolar organoids were constructed using alveolar lavage fluid collected from patients with pulmonary sepsis. The lavage fluid was centrifuged to remove the supernatant, followed by the addition of human peripheral blood lymphocyte separation solution. The cell pellet from the second layer was carefully collected, resuspended in HBSS buffer, and centrifuged again to remove the supernatant. The resulting cell pellet was retained for subsequent processing. An appropriate volume of human alveolar organoid culture medium was added to the cell pellet, proportional to its volume, and the mixture was thoroughly homogenized to create a cell suspension. Matrigel was then added at a 1:1.5 ratio, mixed evenly, and 50 *μ*l of the mixture was pipetted into preheated 24-well cell culture plates. The plates were incubated for 5 min, inverted, and incubated for an additional 20 min to allow the mixture to solidify within the wells. After solidification, 500 *μ*l of human alveolar organoid culture medium was added to each well, and the plates were transferred to a cell incubator. Organoids were cultured at 37 °C in a humidified atmosphere with 5% CO_2_. Organoid growth was monitored daily, with the culture medium replaced every other day to ensure optimal conditions.

### K. Construction of bronchial organoids for pulmonary sepsis in rats

To model pulmonary sepsis *in vitro*, rat bronchial organoids were constructed using bronchi harvested from 5-week-old SPF-grade rats. The bronchi were placed in culture dishes containing Buffer P1 [tissue wash buffer, contained: 500 ml PBS supplemented with 5 ml P1 (CP-00301, ORGEN BIOTECH)] and minced into small fragments using sterile surgical scissors. The fragments were transferred to 15 ml centrifuge tubes, centrifuged to remove the supernatant, and washed twice with Buffer P1, discarding the supernatant after each wash. Tissue digestion solution was added to the centrifuge tubes, and the tubes were placed on a shaker until the tissue fragments appeared flocculent, indicating successful digestion. Digestion was terminated by adding Buffer P2 [cell suspension buffer, contained: 100 ml DMEM/F12 supplemented with 3 ml P2 (CP-00302, ORGEN BIOTECH)]. The resulting suspension, containing numerous cell clusters visible under a microscope, was filtered through a 100 *μ*m cell strainer and centrifuged to remove the supernatant. Red blood cell lysis buffer was added to the cell pellet, incubated at room temperature for 5 min, and centrifuged again to remove the supernatant. The cell pellet was resuspended in Buffer P2, centrifuged once more, and the supernatant was discarded. An appropriate volume of rat bronchial organoid culture medium was added to the pellet, and the mixture was homogenized to form a cell suspension. Matrigel was added at a 1:1.5 ratio, and 50 *μ*l of the mixture was pipetted into preheated 24-well cell culture plates. The plates were incubated for 5 min, inverted, and incubated for an additional 20 min to allow the mixture to solidify. Following solidification, 500 *μ*l of rat bronchial organoid culture medium was added to each well, and the plates were placed in a cell incubator. Organoid growth was monitored daily, with the culture medium replaced every alternate day. Once the rat bronchial organoids were successfully constructed, they were treated with bacterial suspensions (1.628 × 10^9^ CFU/ml *S. aureus* combined with 0.876 × 10^9^ CFU/ml *E. coli*) to generate bronchial organoids mimicking pulmonary sepsis. Following bacterial infection, organoids were co-cultured for 6, 12, and 24 h at 37 °C with 5% CO_2_. Bacterial viability was maintained throughout the infection period. Post-infection analyses included organoid viability assessment using calcein-AM/propidium iodide staining, morphological evaluation, and RNA extraction for gene expression analysis. Both *S. aureus* and *E. coli* strains were procured from the Beijing Beina Chuanglian Biotechnology Institute. All experimental procedures were approved by the Guangxi University of Chinese Medicine Institutional Animal Welfare and Ethical Committee (Approval No. DW20240408-079).

### L. Hematoxylin–eosin (HE) staining and immunofluorescence identification of human alveolar organoids

Mature human alveolar organoids were collected from the culture plates after 12 days of differentiation, when they exhibited stable cystic structures with diameters of 100–200 *μ*m. Mature human alveolar organoids were collected and fixed. After dehydration, clearing, and embedding, paraffin blocks were sectioned and deparaffinized. The sections were stained using an HE staining kit, mounted with neutral resin, and examined under a microscope to evaluate pathological morphological changes in the human alveolar organoids. To identify specific markers, the deparaffinized and blocked sections were incubated overnight at 4 °C with primary antibodies: APQ5 (A9927, Abclonal), SOX9 (82630T, CST), and SPC (A1835, Abclonal). Following three washes with Tris-Buffered Saline with Tween 20 (TBST), the sections were incubated with secondary antibodies for 1 h. Nuclear staining was performed using DAPI for 10 min, followed by three additional washes with TBST. The stained sections were examined under a fluorescence microscope, and images were captured to document the findings.

### M. HE staining and immunohistochemistry identification of rat bronchial organoids

Mature rat bronchial organoids were collected and fixed. After dehydration, clearing, and embedding, paraffin blocks were sectioned and deparaffinized. The sections were stained using an HE staining kit according to the manufacturer's instructions, mounted with neutral resin, and examined under a microscope to assess pathological morphological changes in the rat bronchial organoids. To investigate protein markers, immunohistochemistry was performed on deparaffinized and blocked sections. The sections were incubated overnight at 4 °C with primary antibodies: Ki67 (ab16667, Abcam), P63 (YP-AB-00477, Youpin Biotech), FOXJ1 (Cat# AP69934, Beyotime), MUC5AC (ab3649, Abcam), and CC10 (A16997, Abclonal). Following three washes with PBS, the sections were incubated with secondary antibodies at room temperature for 1 h. Color development was carried out using DAB chromogen, and the reaction was terminated with a rinse in water. The sections were counterstained with hematoxylin for 1 min, followed by rinsing with water, dehydration, and mounting. Microscopic observations and image acquisition were performed to document the findings.

### N. Western blot (WB) and RT-qPCR detection

Proteins from human alveolar organoids were extracted using RIPA lysis buffer and quantified with a BCA protein assay. The extracted proteins were separated by electrophoresis and transferred onto a PVDF membrane. The membrane was blocked and incubated overnight at 4 °C with primary antibodies: ABCC1 (A2223, Abclonal, 1:1000), CYP1B1 (A1377, Abclonal, 1:1000), and PPARG (16643-1-AP, Proteintech, 1:1000). Following three 10-min washes with TBST buffer, the membrane was incubated with the corresponding secondary antibody at room temperature for 1.5 h. After three additional washes with TBST, chemiluminescence (BL520A, Bioss) was utilized for protein detection. Grayscale values of individual protein bands were quantified using ImageJ software, with GAPDH serving as the internal reference to normalize protein expression levels. To further validate the expression of the three key genes, RT-qPCR was performed to quantitatively analyze the mRNA expression levels of *ABCC1*, *CYP1B1*, and *PPARG* in rat bronchial organoids. Table IV presents the primer sequences of *ABCC1*, *CYP1B1*, and *PPARG.*

**TABLE IV.** The primer sequences of *ABCC1*, *CYP1B1*, and *PPARG.*

Primers	Sequence
Abcc1 F	ATGAACATCAGGCAGGAGGAGC
Abcc1 R	AGGTTGACAGAGCCACCAGGAA
Cyp1b1 F	CGGGCATCGCACTTGTACTTC
Cyp1b1 R	TCTCGCCATTCAGCACCACC
Pparg F	CCTTTACCACGGTTGATTTCTC
Pparg R	GGCTCTACTTTGATCGCACTTT

### O. Statistical analysis

MR analysis in this study was conducted using the “TwosampleMR” package in R software (V4.3.2). The inverse variance weighted (IVW) method, recognized for its robustness in causal inference, was employed as the primary approach to evaluate causal associations. To ensure the reliability of the findings, supplemental methods, including MR-Egger, weighted median (WM), simple mode (SM), and weighted mode (WME), were also utilized. Heterogeneity in the MR analysis results was assessed using Cochran's Q test and the I^2^ statistic, while horizontal pleiotropy was evaluated through MR-Egger regression. Sensitivity analysis was performed using the leave-one-out method to determine the influence of individual single-nucleotide polymorphisms (SNPs) on the overall results and to identify potential sources of bias. Causal association results were presented as odds ratios (ORs) with 95% confidence intervals (CIs). An OR > 1 indicated a positive causal association, while an OR < 1 indicated a negative causal association. Bioinformatics and machine learning analyses were also carried out using R software (V4.3.2). For data conforming to a normal distribution, results were expressed as mean ± standard deviation ( $\bar{\text{X}}$ ± s). For data not conforming to a normal distribution, results were presented as quartiles. Group comparisons for normally distributed data with homogeneity of variance were conducted using an independent sample t-test, while a nonparametric rank-sum test was applied to data with non-homogeneous variance or non-normal distributions. Categorical data were expressed as frequencies or percentages, and group comparisons were performed using the chi-square test. A *P*-value <0.05 was considered statistically significant.

## SUPPLEMENTARY MATERIAL

See the supplementary material for expression of Key PANoptosis Genes (*ZBP1*, *PIPK1*, and *PIPK3*, Fig. S1). Figure S2 shows the ROC curve of the model (*ABCC1*, *CYP1B1*, and *PPARG*). PCoA plot (Fig. S3). Pleiotropy of Mendelian randomization in gut microbiota and sepsis (Table S1). 122 types of metabolites that are significantly associated with *Prevotella 9* (Table S2). Detailed information of 24 differentially expressed PFPGs (Table S3). Sensitivity analysis (Table S4). Participant descriptions per cohort in MiBioGen (Table S5). Bray–Curtis dissimilarity matrix (Table S6).

## Data Availability

The data that support the findings of this study are openly available in GEO at https://www.ncbi.nlm.nih.gov/geo/, Ref. 65 and FinnGen at https://www.finngen.fi/en, Ref. 66.
